# A fixed methane filter maximizes freshwater emissions under warming

**DOI:** 10.1038/s41558-026-02649-2

**Published:** 2026-06-05

**Authors:** Sarah F. Harpenslager, Kate Randall, Yizhu Zhu, Michelle C. Jackson, Ian Sanders, Bruno Gallo, Danielle Harris, Hannah Prentice, Yulia V. Bespalaya, Olga V. Aksenova, Alexander Milner, Tom C. Cameron, Boyd A. McKew, Eoin J. O’Gorman, Gabriel Yvon-Durocher, Nikolai Friberg, Kevin J. Purdy, Guy Woodward, Alex J. Dumbrell, Mark Trimmer

**Affiliations:** 1 School of Biological and Behavioural Sciences, Queen Mary University of London, London, UK; 2 B-Ware Research Centre, Nijmegen, the Netherlands; 3 School of Life Science, University of Essex, Colchester, UK; 4 Department of Applied Sciences, Northumbria University, Newcastle, UK; 5 School of Resources and Environment, University of Electronic Science and Technology of China, Chengdu, China; 6 Georgina Mace Centre for the Living Planet, Department of Life Sciences, Imperial College London, Ascot, UK; 7 Department of Biology, University of Oxford, Oxford, UK; 8 N. Laverov Federal Center for Integrated Arctic Research of the Ural Branch of the Russian Academy of Sciences, Arkhangelsk, Russia; 9 School of Geography, Earth and Environmental Sciences, University of Birmingham, Birmingham, UK; 10 Environment and Sustainability Institute, University of Exeter, Penryn, UK; 11 Department of Ecoscience, Aarhus University, Aarhus, Denmark; 12 Freshwater Biological Section, University of Copenhagen, Copenhagen, Denmark; 13 Water@Leeds, School of Geography, University of Leeds, Leeds, UK; 14 School of Life Sciences, Gibbet Hill Campus, University of Warwick, Coventry, UK

**Keywords:** Biogeochemistry, Climate sciences

## Abstract

Approximately half of all methane (CH_4_) emissions come from freshwaters, where they are regulated by the microbial ‘CH_4_ filter’ whose efficiency describes the fraction of CH_4_ produced that is subsequently oxidized back to CO_2_ (methanotrophy) before emission. How the CH_4_ filter efficiency responds to natural warming over centuries or millennia remains unknown. Here we address this question using a natural experiment comprising high-latitude, geothermally warmed streams in five regions spanning the Northern Hemisphere. CH_4_ production becomes more efficient with warming, linked to increased abundance of methanogens and underpinned by community shifts. In contrast, while CH_4_ oxidation activity increases, its process-level efficiency does not, and methanotrophs shift towards less efficient taxa. Consequently, the system-level CH_4_ filter efficiency remains fixed, and CH_4_ emissions increase. If this fixed CH_4_ filter efficiency under warming is common to freshwaters worldwide (wetlands, lakes and rivers), then an upward trajectory for CH_4_ emissions through future climate change appears inevitable.

## Abstract (web summary)

How the balance of microbial methane production and oxidation in freshwater systems will change with warming is unclear. This study uses natural warming experiments to demonstrate that methane emissions increase because oxidation keeps pace with, but cannot exceed, warming-accelerated production.

## Main

**Fig. 1:**
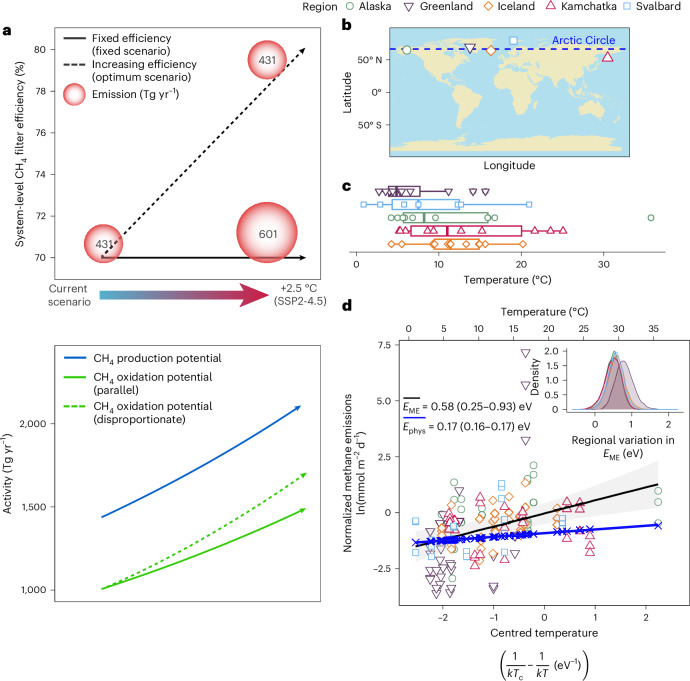
An intercontinental-scale natural experiment to test the effect of warming on the system-level CH_4_ filter efficiency. **a**, The majority of CH_4_ produced (circles, Tg CH_4_ per year) in freshwater sediments and soils is subsequently oxidized by the microbial CH_4_ filter^8,9^, constraining emissions to the atmosphere (current scenario; Supplementary Table 1). If the system-level CH_4_ filter efficiency remains fixed, here at 70%, then under future warming scenarios 2.5 °C (projected for 2100, SSP2-4.5)^48^ CH_4_ production and emissions are predicted to increase 1.4-fold given the recognized temperature sensitivity of CH_4_ production of 0.96 eV (ref. ^10^) (fixed scenario). Only if CH_4_ oxidation increases disproportionately to production will system-level CH_4_ filter efficiency increase, here to 78% under the optimum scenario, to keep future CH_4_ emissions at the current levels^1^ (431 Tg CH_4_ per year), but this has not been tested. **b**, We visited Iceland, Alaska, Greenland, Kamchatka and Svalbard, sampling 10 to 14 streams per region to give >50 streams in total (Methods and Extended Data Fig. 1). **c**, Indirect warming through the bedrock^27,28^ generated a natural warming temperature gradient across all streams from 1 °C to 36 °C. In each box plot, points are individual stream temperatures with the outermost showing the minimum and maximum temperatures across all streams in the region, the box edges show the 25% and 75% quartiles and vertical lines the medians. **d**, Despite regional differences in temperature sensitivities (the inset shows posterior densities for estimated region-level activation energies), CH_4_ emissions (*n* = 148 for 51 streams) increased with temperature across all regions on average (black line; the symbols indicate streams and are shaped and coloured by region; the grey shading shows 95% CIs, also listed in brackets). This increase was faster than expected from physical effects alone (in blue). See Methods for a fuller explanation of the physical effect and ‘Derived quantities and visualization’ for normalized emissions. Basemap data in **b** from Natural Earth (https://www.naturalearthdata.com). Source data

Freshwater wetlands, lakes, rivers and streams are recognized as major sources of the potent greenhouse gas methane (CH_4_) (refs. ^1–4^), but most of the CH_4_ they produce never reaches the atmosphere. Typically, 70–90% of freshwater CH_4_ is oxidized back to the less potent greenhouse gas CO_2_ before emission^5–7^, a process mediated by the microbial ‘CH_4_ filter’^8,9^. Here we define CH_4_ filter efficiency as the fraction of total CH_4_ production that is oxidized prior to emission at the system level. Without this attenuation, CH_4_ emissions from freshwaters would conservatively be more than three times higher than current global estimates^1^ (Fig. 1a, current scenario). This raises a fundamental question: can CH_4_ filter efficiency change under warming, and if so, to what extent might it constrain future CH_4_ emissions?

Methane is produced in anoxic water-logged soils and sediments by specialized microbial archaea (commonly known as methanogens) whose activity and growth increase sharply with warming^10^. Greater CH_4_ production could, in turn, stimulate the CH_4_-oxidizing methanotrophic bacteria^5–7,11,12^, thereby increasing the CH_4_ filter efficiency and constraining emissions^13,14^ (Fig. 1a, optimum scenario). Artificial warming of ponds and peat, however, has shown CH_4_ production to increase beyond that attenuated by the CH_4_ filter, leading to higher emissions^6,15–18^_._ These results suggest that the CH_4_ filter efficiency may be limited, and recent increases in atmospheric CH_4_ have been correlated with warmer wetlands^19,20^. If the CH_4_ filter efficiency is indeed fixed, then higher emissions on a warmer Earth could be inevitable (Fig. 1a, fixed scenario). This fundamental assumption has, however, not been tested in relation to natural warming in freshwaters at large scales under field conditions.

**Extended Data Fig. 1:**
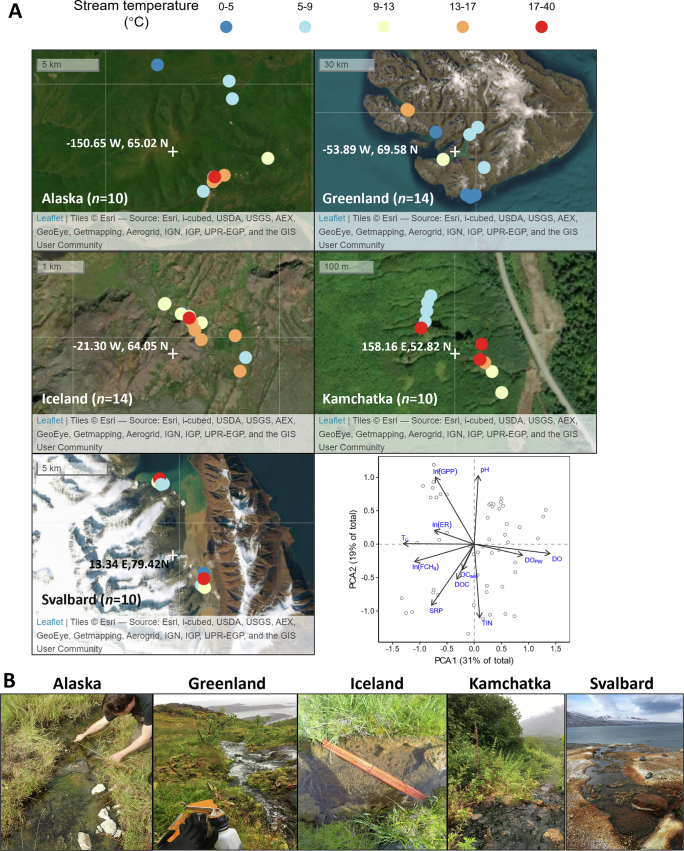
Study regions and stream context. **A**, Each of the five high-latitude regions had a natural thermal gradient nested within it (crosses mark the middle of each map with their longitude and latitude indicated) and **B**, streams were comparable, 1^st^ order, (see Methods and Supplementary Table 3 and 4) with gravel and/or sand sediments. Principal Components Analysis (PCA) identified temperature (Tc) as a key environmental factor in relation to CH_4_ emissions [lnF(CH_4_)]. DOC – dissolved organic carbon; TIN – total inorganic N; SRP – soluble reactive phosphorous; DO – dissolved oxygen, all in stream water. OC_sed_ – organic carbon in streambed sediment; DO_PW_ – porewater oxygen (at 4 cm depth). ER and GPP are ecosystem respiration and gross primary production but are not reported on further here^50,51^. Additional description of the PCA can be found in Extended Data Fig. 2. Aerial maps created in R with leaflet^73^.

**Extended Data Fig. 2:**
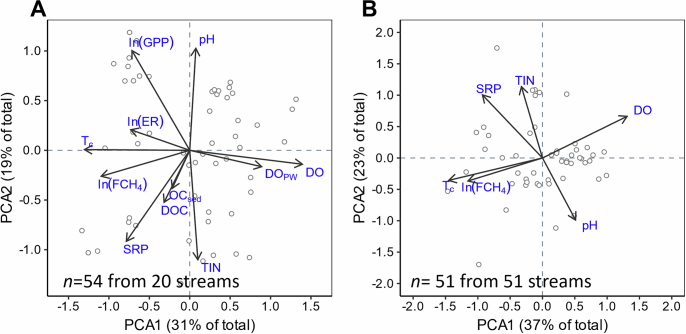
Principal component analysis (PCA) of environmental variables in relation to CH4 emissions. Here we used PCA as quality control for the underlying assumption that we could identify temperature as a key environmental factor to test our specific hypotheses. **A**, for the most complete dataset (*n* = 54 from 20 streams) the ordination explained 50% of the variance in the data, with PCA1 explaining 31% and aligning very well with temperature (centered, T_c_, see equation [12] in the main text). Both CH_4_ emissions (ln[FCH_4_]) and ecosystem respiration (ln[ER]) increased with temperature while gross primary production (ln[GPP]) had a weaker correlation with temperature. Note, we do not report on either ER or GPP any further here as they are not the focus of this study and have, in part, been reported for a subset of the regions elsewhere^50,51^. In contrast, the availability of the major macronutrients that is, C, N, P (organic carbon in sediments [OC_sed_] and dissolved organic carbon [DOC], total inorganic nitrogen [TIN] and soluble reactive phosphorus [SRP] in stream water) were orthogonal to temperature, rather than being confounded with it. The same relationships can be seen in **B**, that, while having fewer variables, is representative of all streams used to measure CH_4_ emissions and here the ordination explained 60% of the overall variance. The strong, positive correlation between CH_4_ emissions and temperature in both **A** and **B** confirmed temperature as a key environmental factor. A subset of the regions in the model system have been used previously to test the effects of temperature on trophic diversity and ecosystem metabolism^49,50^. Note, the negative correlation between T_c_ and stream- water (DO) and porewater-oxygen (DO_pw_) reflect lower O_2_ saturations in warmer water. Porewater could not be collected in Svalbard (see Methods) and the data for Svalbard were omitted in **A**. No physical stream characteristics for example depth, flow or width (Supplementary Table 3) were included as they drive the gas-transfer velocity (*k* m d^−1^) which, in turn, would be confounded with CH_4_ emissions. Source Data

**Extended Data Fig. 3:**
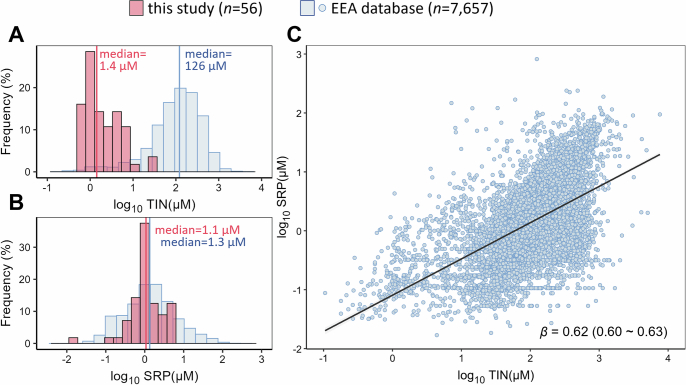
Frequency distributions of total inorganic nitrogen (TIN) and soluble reactive phosphorus (SRP) in the high-latitude streams in comparison to streams in the European Environment Agency (EEA) database cited in Woodward et al.^81^. **A**, with a medium TIN concentration of only 1.4 µM, compared to 126 µM across continental Europe, the streams comprising our natural warming experiment were clearly far removed from any influence of anthropogenic nitrogen. **B**, SRP concentrations were far lower than for TIN and the median of 1.1 µM in our streams represents a natural background imprinted by local geology and weathering. **C**, SRP above this background is indicative of anthropogenic SRP pollution that scales with TIN pollution. Overall, the remoteness of the streams comprising our natural experiment helped identify temperature as a key environmental factor which allowed us to test the effect of warming on CH_4_ production, oxidation and the microbial communities driving the CH_4_ cycle.

Research into the ecological effects of warming generally uses either natural ecosystems along a latitudinal gradient from the poles to the tropics, which is confounded with parallel gradients in biodiversity, light and productivity^21–23^, or artificial experimental warming^17,24–26^, which is restricted both spatially and temporally. Here, to overcome these limitations, we use a unique natural experiment comprising geothermally warmed, headwater streams^27,28^ in five high-latitude regions spanning the Northern Hemisphere (Fig. 1b and Extended Data Fig. 1). Note that the warming here is indirect through the bedrock, which is distinct from that in, for example, Yellowstone National Park, which experiences extreme water chemistries^27^. Each region has its own ambient-to-warmed temperature gradient, and together the intercontinental range is 1–36 °C (Fig. 1c). Being high-latitude, the streams are free from anthropogenic pollution (sewage, fertilizer and excess sediments), meaning that temperature is a key environmental factor explaining structure in the data (principal components analysis; Extended Data Figs. 1–3). This natural experiment allows us to test the effects of warming on key components of the CH_4_ cycle at an unprecedented spatial scale and after many more microbial generations than captured by experiments to date^15,17,24,29^. We test the hypothesis that the system-level CH_4_ filter efficiency remains fixed under warming, such that CH_4_ emissions increase. For emissions to be constrained, CH_4_ oxidation would need to increase disproportionately relative to CH_4_ production (Fig. 1a). We also test whether warming alters the composition of the underlying methanogen and methanotroph communities^15,29^. Although our natural experiment is based on high-latitude streams, the principle that CH_4_ emissions are governed by the relative responses of production and oxidation—and thus by the CH_4_ filter efficiency—provides a proxy for understanding the wider implications of warming on CH_4_ emissions across freshwaters more generally^1–4^.

## Consistent increase in CH_4_ emissions with warming

**Extended Data Fig. 4:**
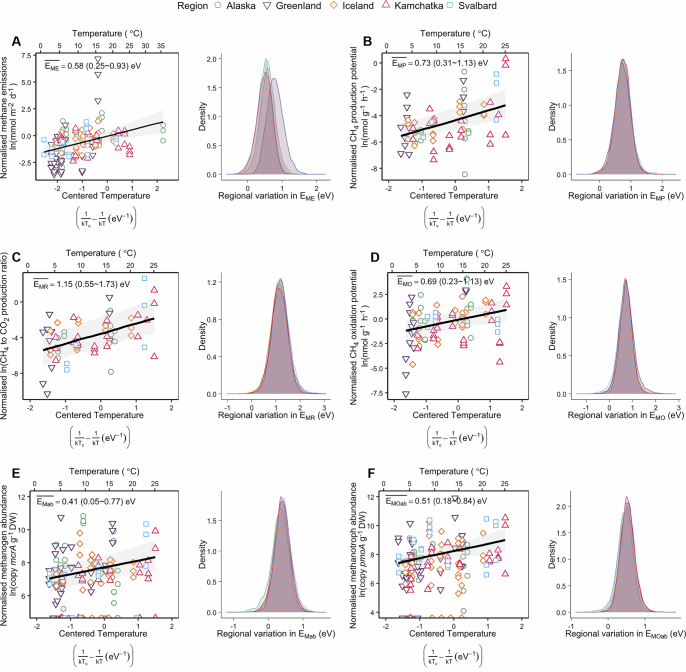
Methane emission (A), CH4 production potential (B), CH4 to CO2 production ratio (C), CH4 oxidation potential (D), methanogen abundance (E) and methanotroph abundance (F) increased in warmer streams across all regions despite regional differences in their temperature sensitivities. The overall temperature sensitivities across the five regions were estimated by fitting Bayesian Linear Mixed Effects Models (see Methods) and the density plots demonstrate variation across regions in the posterior distributions of temperature sensitivities. Symbols are streams shaped and coloured by region and display capacity normalised responses, grey shading shows 95% CIs. Source Data

**Extended Data Fig. 5:**
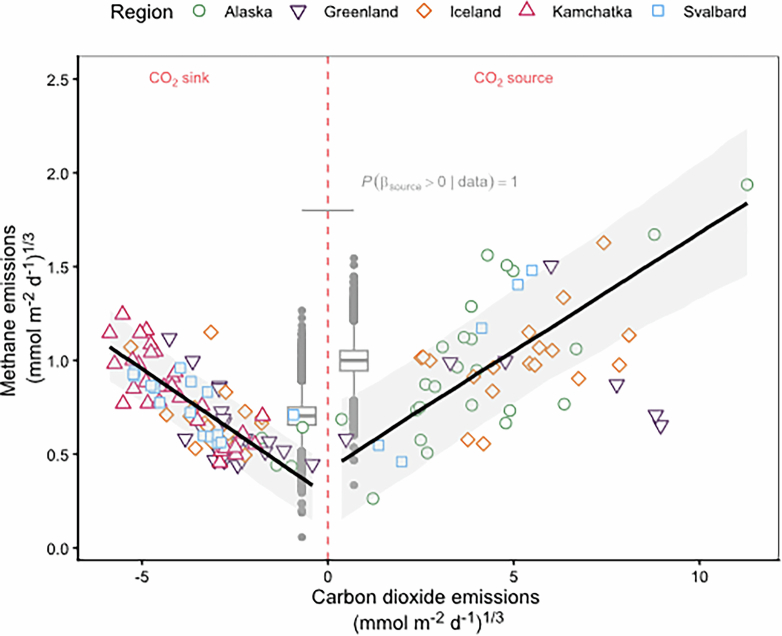
Some streams when sampled were acting as net sinks for CO2 and others as net sources with CH4 emissions correlated with both (scatter-plots, n = 148 for 51 streams). Although CH_4_ emissions were higher overall from CO_2_-source streams (1.0 mmol CH_4_ m^−2^ d^−1^, *P*(*β*_source_ > 0|data)=1, *β*_source_ is the effect of warm streams relative to the cold) than from CO_2_-sink streams (0.7 mmol CH_4_ m^−2^ d^−1^), likely reflecting differences in organic substrate availability^3,31^, the temperature sensitivities of their emissions were comparable (*E*_ME_ = 0.52 eV, 95% CI: 0.18 to 0.87 eV in CO_2_-sink streams while *E*_ME_ = 0.58 eV, 95% CI: 0.10 to 1.05 eV in CO_2_-source streams). Black lines are correlations between methane emissions and carbon dioxide emissions fitted using Bayesian Linear Mixed Effects Models (symbols are streams shaped and coloured by region; grey shading shows 95% CI). Data have been cube-root^1/3^ transformed to improve normality while preserving the negative and positive structure of the original data. Box-plots denote 25% and 75% quartiles, medians and interquartile ranges of posterior distributions. Source Data

Rivers and streams worldwide emit CH_4_ at a comparable magnitude to lakes (28 Tg CH_4_ per year versus 42 Tg CH_4_ per year)^3,4^, with variation in emission correlated with multiple biotic and abiotic factors^3,30^. Here we found a clear temperature sensitivity for CH_4_ emissions (*E*_ME_ = 0.58 eV; 95% credible interval (CI), 0.25 to 0.93 eV) with consistently higher emissions from warmer streams, on average (Fig. 1d, Supplementary Table 5 and Extended Data Fig. 4). The average activation energy for CH_4_ emissions we observed across all five regions is consistent with temperature sensitivities found in meta-analyses of CH_4_ emissions from 127 lakes, rivers and wetlands globally^10^. We also found substantial region-to-region differences (0.50 to 0.79 eV; Fig. 1d, inset) in the activation energy of CH_4_ emissions that probably represent differences in hydrology, geomorphology, organic matter and their covariance with temperature across the gradients in each of the regions. In contrast to CH_4_, some streams when sampled were acting as net sinks for CO_2_ and others as net sources (Extended Data Fig. 4). Although CH_4_ emissions were higher overall from CO_2_-source streams (1.0 mmol CH_4_ m^−2^ d^−1^) than from CO_2_-sink streams (0.7 mmol CH_4_ m^−2^ d^−1^), probably reflecting differences in organic substrate availability^3,31^, the temperature sensitivity of their emissions was comparable (Extended Data Fig. 5).

Even without any increase in CH_4_ production, CH_4_ emissions would be expected to rise in warmer streams^10,32^, due to higher gas-transfer velocities and lower CH_4_ solubility at elevated temperatures^33^. To account for this, we used the average measured CH_4_ concentration across all streams, in combination with gas-transfer velocities and CH_4_ saturation—both scaled to in situ temperature—to simulate the physical increase (Methods). Our measured increase in CH_4_ emissions was about fourfold greater (for example, *E*_ME_ = 0.58 eV versus *E*_phys_ = 0.17 eV; Fig. 1d) than any rise due to physical effects alone, which indicated elevated CH_4_ production in the warmer streambed sediments.

## Methane production efficiency increases with warming

**Fig. 2:**
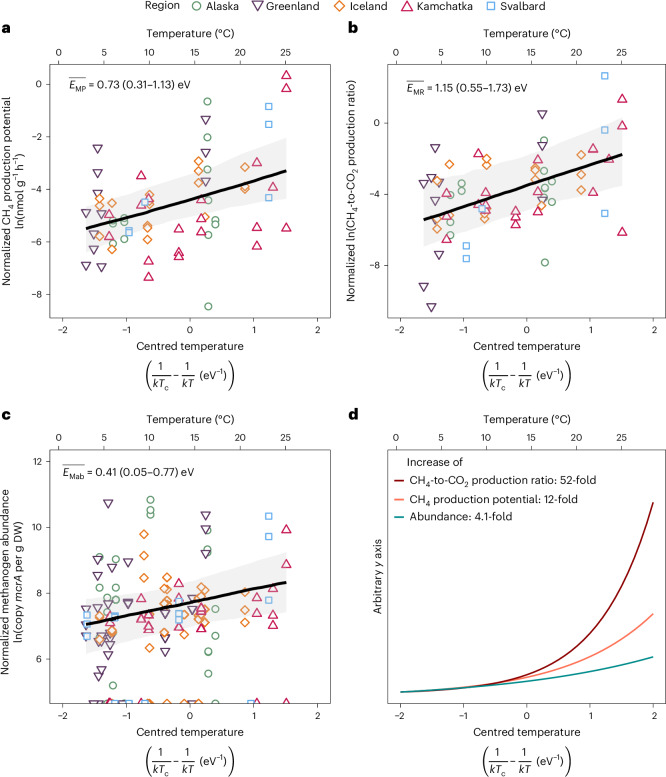
Sharp increase in streambed CH_4_-to-CO_2_ production potentials alongside a gentler rise in methanogen abundance. **a**,**b**, Streambed CH_4_ production potential increases with a common temperature sensitivity (*E*_MP_ = 0.73 eV; black line; the 95% CI is shown in brackets; *n* = 71 for 28 streams) across the five regions (symbol shapes and colours) (**a**), but not as rapidly as the increase in CH_4_:CO_2_ production ratio (*E*_MR_ = 1.15 eV, as for **a**) (**b**). **c**, The increases in **a** and **b** were only partly reflected in an increase in methanogen abundance (*E*_Mab_ = 0.41 eV; as for **a**; *n* = 156 for 52 streams in five regions; zero *mcrA* copy numbers were accounted for using a zero-inflated model and are demonstrated separately at the bottom to reflect their frequency). DW, dry weight. **d**, Illustrative comparison of the predicted, exponential increases in CH_4_ production, CH_4_:CO_2_ and methanogen abundance between 5 °C and 30°C. The exponents in **a** (0.73), **b** (1.15) and **c** (0.41) are used in **d**, but the starting point (intercept) for each has been set to 0, and the *y* axis is arbitrary. In **a**–**c**, the symbols display capacity-normalized responses centred at their region-specific log capacity at *T*_c_, and the black lines indicate the average temperature effect with 95% CIs in grey shading, also listed in brackets. See ‘Derived quantities and visualization’ in Methods. Source data

The temperature sensitivity of CH_4_ production is well characterized across microbial assemblages to whole-ecosystem levels of organization (that is, 0.96 eV)^10^, and our measured increase in CH_4_ production potential (12-fold) as streambed sediments warmed was in line with previous studies (Fig. 2a; 0.73 eV across all regions; 95% CI, 0.31 to 1.13 eV). In contrast, the much greater 52-fold increase in the CH_4_:CO_2_ production ratio (Fig. 2b; 1.15 eV across all regions; 95% CI, 0.55 to 1.73 eV) suggested improved substrate use alongside the physiological response to temperature. Whereas the increase in methanogen abundance (Fig. 2c; 0.41 eV across all regions; 95% CI, 0.05 to 0.77 eV), coupled with higher temperatures, could explain the 12-fold increase in CH_4_ production potential, the disproportionate rise in the CH_4_:CO_2_ ratio pointed to more effective substrate use (Fig. 2d)^34^.

**Extended Data Fig. 6:**
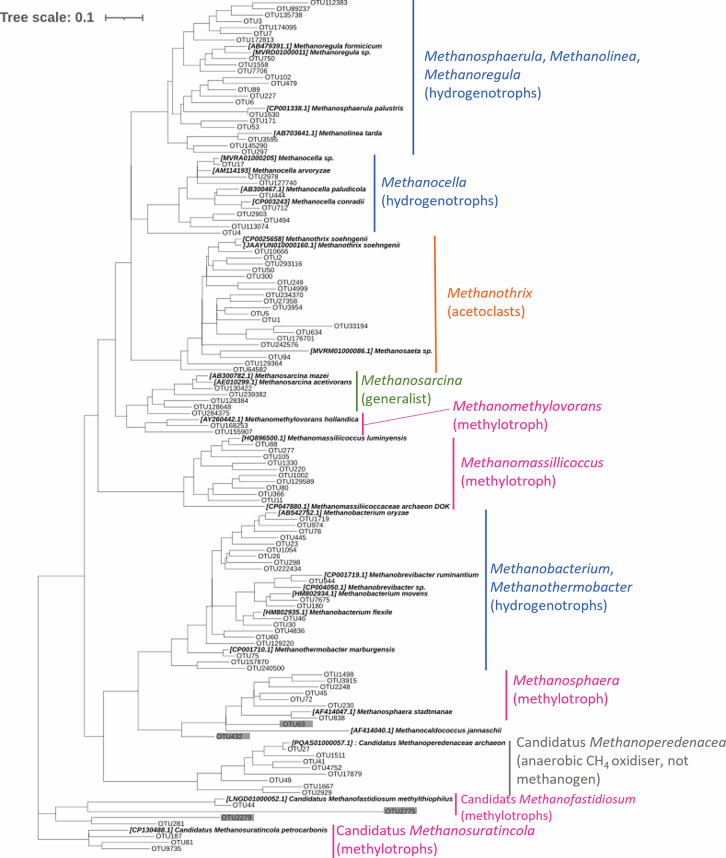
Phylogenetic tree of 110 mcrA OTUs constructed from 27 reference sequences of known methanogen cultures. Based on the phylogenetic placements of *mcrA* OTUs on the tree with the taxonomy of reference sequences (marked in bold and italics), we were able to assign taxonomic classification then annotate the methanogenic pathways to the majority of the OTUs (93 OTUs from 3,793,456 reads out of a total 110 OTUs from 4,199,328 reads, that is 90 % in terms of reads count). We took this as good evidence that our sequencing library captured the overall characteristics of the methanogen community in the streambed sediments. Therefore we used the 93 OTUs from 3,793,456 reads as the representative *mcrA* community within which there were: 19 OTUs as acetoclasts (1,160,280 reads, at 30.5% of the *mcrA* community, within *Methanothrix* solely)^82^; 50 OTUs as hydrogenotrophs (2,292,004 reads, at 60.2% of the total *mcrA* library, within genera *Methanosphaerula*^83^, *Methanolinea*^83^*, Methanoregula*^83^*, Methanocella*^83,84^*, Methanobacterium*^85,86^*, Methanobrevibacter*^87,88^ and *Methanothermobacter*^89^); and 24 OTUs as methylotrophs (341,172 reads, at 9% of the total *mcrA* library, within *Methanomethylovorans*^90^, *Methanolobus*^91^, *Methanosphaera*^92^*, Methanomassiliicoccus*^93^, Candidatus *Methanosuratincola*^94^ and Candidatus *Methanofastidiosum*^95,96^), respectively. Note that the publications supporting the annotation of methanogenic pathways are cited by genus. Two genera (*Methanobacterium* and *Methanobrevibacter*) contain species that may utilise alcohol-like substrates, however, this does not reclassify the hydrogenotrophic methanogen phyla we detected in our streams as methylotrophs. *Methanobacterium bryantii* and *Methanobacterium formicicum* can use H_2_ + CO_2_ and secondary-alcohols as substrates^84^, but none of the OTUs detected in the streambed sediments were closely related to these species. *Methanobrevibacter ruminantium* and *Methanobrevibacter* AbM4 contain the genes related to alcohol utilization^97,98^, however, the genus *Methanobrevibacter* are still widely recognized as hydrogenotrophic methanogens^87,88^. Also, note that 8 OTUs from 405,872 reads were removed for being assigned as the non-methanogen taxon (Candidatus *Methanoperedenacea*)^99^ and an additional 9 OTUs from 13,891 reads (shaded in grey) were also removed for being either assigned as generalist methanogens (*Methanosarcina*)^100^ or for being placed outside of clades for any known references. Source data

In freshwater soils and sediments, hydrogenotrophic, acetoclastic and methylotrophic methanogens use fermentation products (for example, acetate, CO_2_, H_2_ and methyl compounds) to generate CH_4_ and CO_2_ (Supplementary Table 2). As methyl compounds are relatively scarce^35–37^, methanogenesis is usually dominated by hydrogenotrophs and acetoclasts, which under optimal conditions can yield CH_4_ and CO_2_ in a 1:1 ratio^34,38^ (Supplementary Table 2 and Extended Data Fig. 6). In practice, however, ratios closer to 0.1:1 are typical, reflecting inefficient substrate use, which is known to improve at higher temperatures^34,38^. In our study, the CH_4_:CO_2_ production ratio—and by inference methanogenic substrate use—increased 52-fold, from 0.005:1 in the coldest streams to 0.26:1 in the warmest (Fig. 2d)^34,38^. Increases in the fraction of any of the available substrates (for example, acetate, H_2_ or methyl compounds) used by methanogens could have driven up the ratio of CH_4_:CO_2_ production. This disproportionate increase suggests that warming enhanced substrate use. If substrate use had increased equally across all pathways, we would have expected methanogen community composition to remain conserved, but this was not what we observed.

**Fig. 3:**
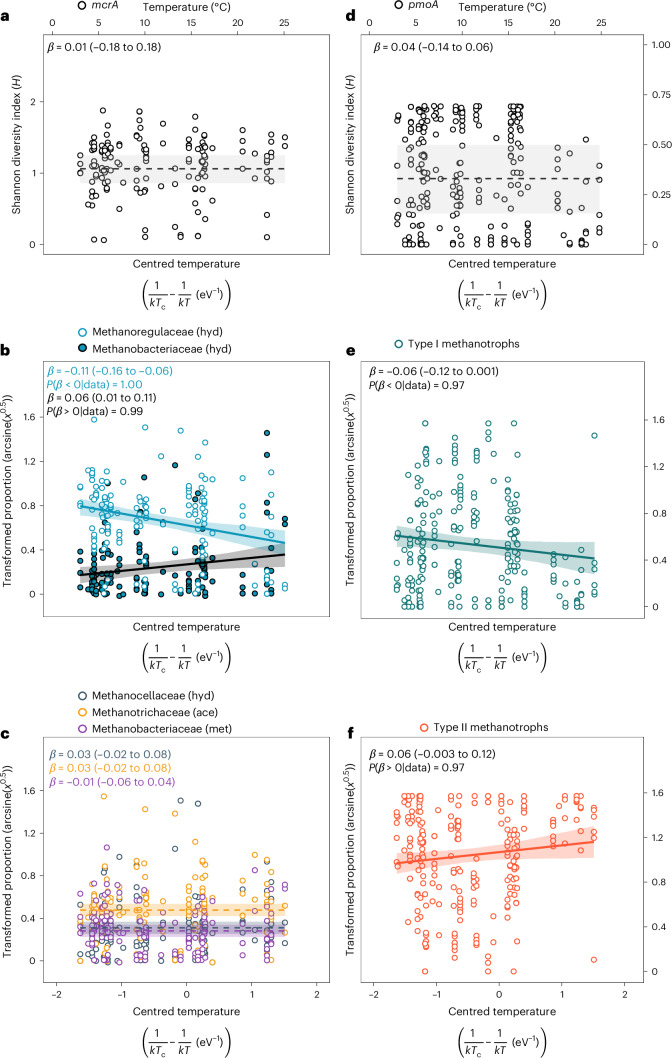
Composition of CH_4_-producing and CH_4_-oxidizing microbial communities along a natural warming gradient. **a**,**b**, The diversity of methanogens was conserved (**a**), but there was sorting of the two hydrogenotrophic (hyd) families, Methanoregulaceae and Methanobacteriaceae (**b**). **c**, Relative abundance of other methanogen families—hydrogenotrophic Methanocellaceae (hyd), acetoclastic Methaotrichaceae (ace) and the grouped methylotroph families (met)—did not change. **d**–**f**, Overall diversity of methanotrophs was conserved (**d**), but the relative abundance of type I methanotrophs decreased while type II increased with warming (**e**,**f**). The symbols and colours only distinguish between families and functional groups (*n* = 156 and 251 for the methanogen and methanotroph communities, respectively, for 52 streams across the five regions). The proportions in **b**, **c**, **e** and **f** were arcsine-transformed to improve normality. The solid lines in **b**, **e** and **f** represent average temperature effects with strong Bayesian evidence (slope (*β*), posterior probability > 0.95; Supplementary Table 7). The dashed lines in **a**, **c** and **d**, where 95% CIs for *β* cross over zero, indicate the overall average estimates of relative abundance (intercepts). The shaded areas show 95% CIs. Source data

Our streambed methanogen communities were dominated (~91%) by hydrogenotrophs and acetoclasts (60.2% and 30.5% of the *mcrA* metabarcoding reads, respectively; Extended Data Fig. 6), consistent with freshwater sediments^38,39^. While overall methanogen diversity remained constant with warming (*β* = 0.01; 95% CI, –0.18 to 0.18; Fig. 3a), we observed significant changes between two hydrogenotroph families. Specifically, as the proportion of Methanobacteriaceae increased (*β* = 0.06; 95% CI, 0.01 to 0.11; *P*(*β* > 0|data) = 0.99; one-sided hypothesis test), Methanoregulaceae decreased (*β* = −0.11; 95% CI, −0.16 to −0.06; *P*(*β* < 0|data) = 1.00; one-sided hypothesis test; Fig. 3b), while acetoclasts (Methanotrichaceae), methylotrophs (four families grouped) and other hydrogenotrophs (Methanocellaceae) were conserved (95% CIs include zero; Fig. 3c). Warming is known to facilitate greater H_2_ use by hydrogenotrophs to produce CH_4_ (ref. ^40^), but here it appeared that not all hydrogenotrophs exploited this advantage. The relative increase in Methanobacteriaceae suggested they used H_2_ more efficiently than Methanoregulaceae or Methanocelleceae^41^ as temperatures rose. Methanobacteriaceae were therefore probably responsible for the higher CH_4_:CO_2_ production ratio (Fig. 2d) and more efficient CH_4_ production potentials in warmer streambeds (that is, more H_2_ from reaction 1 was used to reduce CO_2_ to CH_4_ through reaction 2; Supplementary Table 2). In earlier field mesocosm experiments, we showed that 11 years of +4 °C warming drove subtle shifts in hydrogenotrophs that correlated with increased CH_4_ emissions^15^. Here we observed similar but much stronger responses across multiple regions spanning the Northern Hemisphere.

## System-level filter efficiency remains fixed under warming

**Fig. 4:**
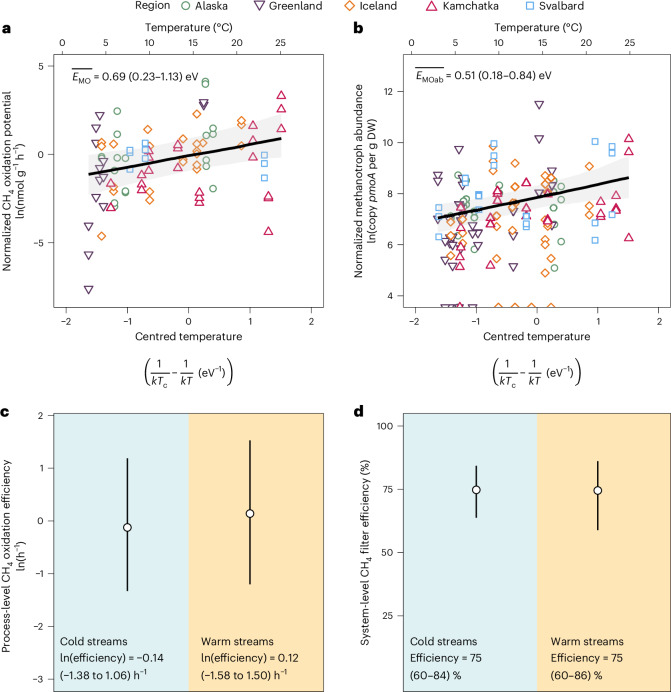
Streambed CH_4_ oxidation potential and methanotroph abundance increase with warming while CH_4_ filter efficiency remains fixed. **a**,**b**, Sharp increase in CH_4_ oxidation potential (E_MO_ = 0.69; black line; 95% CI is shown in brackets; *n* = 89 for 32 streams in five regions) (**a**) and methanotroph abundance (E_MOab_ = 0.51; as for **a**; *n* = 156 for 52 streams in five regions, and see Fig. 2 for an explanation of the different sample sizes; zero *pmoA* copy numbers were accounted using a zero-inflated model and are demonstrated separately at the bottom to reflect their frequency) (**b**) in warmer streams, on par with the parallel increases in both CH_4_ production and methanogen abundance. **c**,**d**, In contrast, the process-level oxidation efficiency (porewater CH_4_ turnover rate per hour; equation (10)) remained fixed (the vertical bars indicate overlapping 95% CIs in cold and warm streams; *n* = 73 for 27 streams in four regions) (**c**), which was reflected in the system-level CH_4_ filter efficiency (equation (11)) being fixed at 75% in both our relatively cold and warm streams (overlapping 95% CIs; *n* = 70 for 26 streams in four regions) (**d**). In **a** and **b**, the symbols indicate streams, are shaped and coloured by region, and display capacity-normalized responses centred at their region-specific log capacity at *T*_c_; the black lines are the average temperature effect with 95% CIs in grey shading. See ‘Derived quantities and visualization’ in Methods. The data in **c** and **d** were grouped as streams above or below the median stream temperature of 10.5 °C as either ‘warm’ or ‘cold’, respectively. Source data

In line with the patterns for CH_4_ production potential and methanogen abundance (Fig. 2), CH_4_ oxidation potential (Fig. 4a; *E*_MO_ = 0.69 eV; 95% CI, 0.23 to 1.13 eV) and methanotroph abundance (Fig. 4b; *pmoA* gene copy number, *E*_MOab_ = 0.51 eV; 95% CI, 0.18 to 0.84 eV) increased in warmer streambed sediments. Despite this, the process-level oxidation efficiency did not respond to warming (Fig. 4c; that is, the turnover rate for porewater CH_4_ per hour; equation (10) in Methods). Thus, although CH_4_ oxidation increased in warmer sediments, it only kept pace with warming-induced increases in CH_4_ production (Fig. 2a) and did not exceed it (Fig. 1a, fixed versus optimum scenario); that is, higher CH_4_ production simply provided more substrate to oxidize. This process-level constraint was reflected in our measure of the system-level CH_4_ filter efficiency (equation (11) in Methods).

For small headwater streams like those studied here, the streambed dominates the supply of CH_4_ to the overlying water column^42,43^_._ The ratio of streambed to stream water CH_4_ therefore provides a relative measure of the system-level CH_4_ filter efficiency, since CH_4_ produced in the streambed must pass through the CH_4_ filter before reaching the overlying water. This ratio showed that the system-level CH_4_ filter efficiency was fixed at ~75% in both colder and warmer streams (Fig. 4d). These fixed process-level and system-level efficiencies together explain why CH_4_ emissions increased in warmer streams (Fig. 1d). Given that we had observed changes in hydrogenotrophic methanogens associated with higher CH_4_ production, we next examined whether the composition of the methanotroph community could explain the fixed CH_4_ filter efficiency.

**Extended Data Fig. 7:**
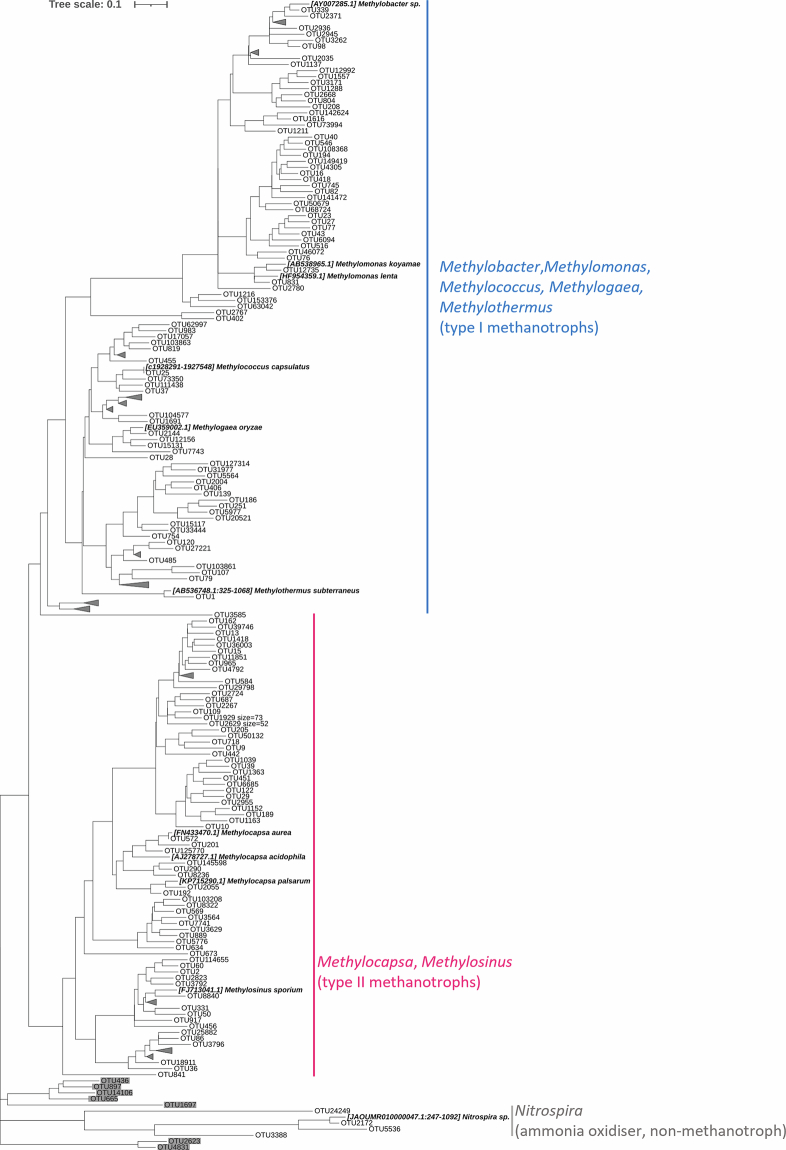
Phylogenetic tree of 264 pmoA OTUs constructed from 17 reference sequences of known methanotroph cultures. Traditionally, methanotrophs are divided into two groups, that is, type I methanotrophs (γ-Proteobacteria) and type II methanotrophs (α-Proteobacteria). In this study, as we intended to test for any shift in the methanotroph community with warming in terms of their types (Fig. 3,e and f), we did not attempt to assign taxonomy classification but to annotate methanotrophic types to each *pmoA* OTU only. Based on the phylogenetic placement of OTUs on the tree with the taxonomy of the reference sequences (marked in bold and italics), the main two methanotrophic types formed two distinct clades thus we were able to annotate 159 OTUs as type I methanotrophs (735,301 reads, including genera *Methylobacter*, *Methylomonas*, *Methylococcus*, *Methylogaea* and *Methylothermus*)^8,101,102^ and 94 OTUs as type II (1,409,349 reads, including *Methylocapsa* and *Methylosinus*)^103^. These two groups together constituted 99.9 % of the total of 2,146,954 reads sequenced from the streambed sediments. Therefore, like the phylogenetic classification performed to the methanogen OTUs (Extended Data Fig. 6), we took this as good evidence that our sequencing library captured the overall characteristics of the methanotroph community. Note, 4 of the OTUs from 453 reads were removed as they were assigned as non-methanotroph taxon (*Nitrospira*)^104^ and an additional 7 OTUs from 1,851 reads (shaded in grey) were also removed for falling outside any clades of any known references. To facilitate visualisation, clades with bootstrap values of <0.08 similarity were collapsed. Source data

Traditionally, CH_4_ oxidizers are categorized as type I ‘high-efficiency’ and type II ‘low-efficiency’ methanotrophs^13,14,44^. Type I are often prevalent in CH_4_-rich, low-oxygen environments, where a high type I:II ratio enhances filter efficiency and mitigates emissions^13,14^. We therefore anticipated that the higher CH_4_ (~400 nM versus 200 nM) and lower oxygen (~250 μM versus ~350 μM) in warmer streams would favour type I over type II (Extended Data Fig. 7). However, overall diversity was conserved (*β* = 0.04; 95% CI, –0.14 to 0.06; Fig. 3d), but with warming the relative abundance of type I decreased (*β* = −0.06; 95% CI, −0.012 to 0.001; *P*(*β* < 0|data) = 0.97; one-sided hypothesis test; Fig. 3e), while low-efficiency type II increased (*β* = 0.06; 95% CI, −0.003 to 0.12; *P*(*β* > 0|data) = 0.97; one-sided hypothesis test; Fig. 3f). Although species-level effects are recognized in some methanotrophs^45^, the broader pattern suggests that warming, rather than CH_4_ availability, favoured type II methanotrophs, as has been reported widely in paddy and forest soils^14,46^. In our natural experiment, such warming-induced sorting of the methanotroph community probably contributed to the system-level CH_4_ filter efficiency remaining fixed, and thus to higher CH_4_ emissions with warming. These intercontinental-scale observations strongly cross-validate earlier reports of increased CH_4_ emissions from experimentally warmed ponds^15,24^ and peat^41^, demonstrating their broader relevance across the natural world^47^.

## Conclusions

Recent increases in atmospheric CH_4_ have been linked to higher emissions from warmer wetlands, yet the complete underpinning mechanisms have not been tested in natural systems^19,20^. Our natural experiment shows that even after acclimation and/or adaptation, over many more microbial generations than previously captured experimentally^17,24–26^, CH_4_ oxidation could only keep pace with but not exceed the increases in CH_4_ production induced by natural warming. As a result, the system-level CH_4_ filter efficiency remained fixed and so could not constrain emissions under warming. While previous datasets demonstrated parallel temperature sensitivities for emissions and production across ecosystems^10^, our natural experiment extends this to multiple levels of organization—from methanogen and methanotroph abundance, to production, oxidation and emissions—in response to warming, across the Northern Hemisphere (Extended Data Fig. 4). Because CH_4_ emissions are governed by the relative responses of production and oxidation, this principle is likely to apply across freshwater wetlands, lakes, rivers and peatlands^1–4^ even where production and ebullition are higher (Supplementary Discussion). If the fixed efficiency of the CH_4_ filter we observed proves widespread, then an upward trajectory of CH_4_ emissions under future climate warming appears inevitable.

## Methods

### Field sites

Over the course of two summers, 2016 and 2017, we visited geothermal catchments in five high-latitude regions across the Northern Hemisphere: Hengill Valley in Iceland; Manley Hot Springs in Alaska; Disko Island in Greenland; the North-Western Spitsbergen National Park in Svalbard; and the Verkhne-Paratunskiye thermal springs in Kamchatka, Russia (Fig. 1b, Extended Data Fig. 1 and Supplementary Table 3). Here, indirect warming through the bedrock^27,28^ generated a natural warming gradient in the streams from 1 °C to 36 °C, and we typically sampled 10 to 14 streams per region to give >50 streams in total (Fig. 1b,c and Extended Data Fig. 1; for further detailed site descriptions, see refs. ^27,28^). The streams were all first order with gravel and/or sand bed sediments, were far from any anthropogenic influence, were circumneutral and shared comparable hydrophysical and chemical characteristics (Extended Data Fig. 3 and Supplementary Tables 3 and 4). Some regions of the natural experiment have been used previously to test the effect of temperature on trophic diversity^49^ and ecosystem respiration^50,51^.

### Stream characteristics

Hydrophysical and chemical properties of the streams were determined within 50-m transects. At each stream, a transect of ten equally spaced sampling locations was established, and at three equally spaced locations along the transect, measurements were recorded for stream flow velocity (m s^−1^). At one upstream location, the pH and dissolved oxygen concentration in stream water were measured using sensors connected to a multiprobe^52^ (a PHC301 pH electrode and an INTELLICAL Standard LDO sensor, respectively, connected to a Hach HQ40D multimeter). Loggers were also deployed in each stream to continuously record temperature (MiniDot, RS Aqua) for a minimum of 24 hours (average four to five days in each stream).

Sediment porewater probes^53^ were inserted at 2, 4, 6, 8 and 10 cm depth where streambeds were penetrable, giving us five in Alaska, five in Greenland, eight in Iceland and eight in Kamchatka but none in Svalbard, where the streambed was armoured with cobbles^54^. Porewater (*n* = 3 for each depth) was drawn up using a 30-ml syringe, immediately transferred to a gas-tight vial (3 ml, Exetainer, Labco) and fixed with 15 µl of ZnCl_2_ (7 M) for later analysis of dissolved CH_4_ back in the laboratory at Queen Mary University of London, as described below.

### Estimating in situ CH_4_ and CO_2_ emissions from streams to the atmosphere

Surface water samples for dissolved gases were collected at three equally spaced locations along each stream transect and preserved (as above) for later quantification of dissolved CH_4_ and CO_2_ (after catalytic reduction by hot nickel to CH_4_) using a gas chromatograph fitted with a flame ionization detector as described before^55^. Total concentrations (*C*_measured_) per vial (headspace and water) for each gas were calculated using the respective Bunsen coefficients and Henry’s law^56,57^. Saturation at atmospheric equilibration (*C*_saturation_) for each gas in each stream was determined using temperature and salinity data and the global atmospheric concentrations (CDIAC_GOV_Updated 2016, http://cdiac.ess-dive.lbl.gov/pns/current_ghg.html). The emission of CH_4_ or CO_2_ was calculated using the following equation:

1 $$F=({C}_{{\rm{measured}}}-{C}_{{\rm{saturation}}})\times {k}_{{\rm{gas}}{\rm{\_}}(T)}$$

where *F* is the emission of CH_4_ or CO_2_ (mmol m^−2^ d^−1^), *C*_measured_ is the measured concentration of CH_4_ or CO_2_ in stream water (mmol l^−1^; for CO_2_ derived from pH and total DIC (dissolved inorganic carbon) after acidification; equations (7) and (8)) and *C*_saturation_ is the concentration of CH_4_ or CO_2_ at atmospheric equilibration (mmol l^−1^) as above. The degree of oversaturation for CH_4_ in the streams (*C*_measured_/*C*_saturation_) is shown in Supplementary Fig. 4. *k*_gas_(*T*)_ is the gas transfer velocity for CH_4_ or CO_2_ (m d^−1^) at in situ temperature *T* derived from *k*_O2_20°__C_ (m d^−1^) and the appropriate Schmidt numbers as follows:

2 $${k}_{{\rm{O}}2{{\_}}{20^\circ}{\rm{C}}}=\frac{50.8\times {(u\times 100)}^{0.67}}{{\left(d\times 100\right)}^{0.85}/100}\times 24$$

3 $${\rm{Sc}}=A-B\times T+C\times {T}^{2}-D\times {T}^{3}$$

4 $${k}_{\mathrm{CH}4{\rm{\_}}20^\circ {\rm{C}}}={k}_{{\rm{O}}2{\rm{\_}}20^\circ {\rm{C}}}\times {\left(\frac{{\mathrm{Sc}}_{\mathrm{CH}4}}{{\mathrm{Sc}}_{{\rm{O}}2}}\right)}^{-2/3}\mathrm{or}{k}_{\mathrm{CO}2{\rm{\_}}20^\circ {\rm{C}}}={k}_{{\rm{O}}2{\rm{\_}}20^\circ {\rm{C}}}\times {\left(\frac{{\mathrm{Sc}}_{\mathrm{CO}2}}{{\mathrm{Sc}}_{{\rm{O}}2}}\right)}^{-2/3}$$

5 $${k}_{{\rm{CH}}4{\rm{\_}}({\rm{T}})}={k}_{{\rm{CH}}4{\rm{\_}}20^\circ {\rm{C}}}\times {1.025}^{(T-20)}{\rm{or}}{k}_{{\rm{CO}}2{\rm{\_}}({\rm{T}})}={k}_{{\rm{CO}}2{\rm{\_}}20^\circ {\rm{C}}}\times {1.025}^{(T-20)}$$

where *k*_O2_20°__C_ (m d^−1^) is the gas transfer velocity for dissolved O_2_ calculated from stream velocity (*u*, m s^−1^) and depth (*d*, m). This equation is applicable to streams with velocities ranging from 3 cm s^−1^ to 150 cm s^−1^ (ref. ^58^). Given that 90% of our streams fell within this velocity range (3 cm s^−1^ and 76 cm s^−1^ at the 5% and 95% percentiles, respectively), we considered equation (2) appropriate for estimating the gas transfer velocity for this study. Sc is the Schmidt number for CH_4_ (Sc_CH__4_), CO_2_ (Sc_CO__2_) or O_2_ (Sc_O__2_) calculated from their corresponding sets of coefficients (for CH_4_, *A* = 1,897.8, *B* = −114.28, *C* = 3.2902 and *D* = −0.0391; for CO_2_, *A* = 1,911, *B* = −118.11, *C* = 3.453 and *D* = −0.0413; and for O_2_, *A* = 1,800.6, *B* = −120.1, *C* = 3.7817 and *D* = −0.0476) at in situ temperature *T* (°C)^33^. *k*_O2_20°__C_ was subsequently specified for CH_4_ or CO_2_ using the ratio of their Schmidt numbers to that of O_2_ and scaled to in situ temperature *T* (ref. ^59^). Finally, the specified *k*_CH4_(*T*)_ and *k*_CO2_(*T*)_ were used to calculate emissions of CH_4_ or CO_2_ in equation (1).

### Simulated CH_4_ emissions due to physical effects

To compare any measured increase in CH_4_ emission to that driven purely by any increase in physical processes in warmer streams (that is, higher gas transfer velocity and lower gas saturations), we simulated physical CH_4_ emissions using:

6 $${F}_{{\rm{sim}}}=({{C}^{{\prime} }}_{{\rm{measured}}}-{C}_{{\rm{saturation}}})\times {{k}^{{\prime} }}_{{\rm{gas}}{\rm{\_}}({\rm{T}}){\rm{\_}}{\rm{med}}}$$

where *F*_sim_ is the simulated physical CH_4_ emission (mmol m^−2^ d^−1^), *C*′_measured_ is the median of all measured CH_4_ concentrations in all our streams (mmol l^−1^, *n* = 51 streams) and *C*_saturation_ is the concentration of CH_4_ at atmospheric equilibration (mmol l^−1^) at stream temperature, as above. *k*′_gas_(*T*)_ is calculated from the median of *k*_O2_20°__C_ derived in equation (2) and scaled to CH_4_ at in situ temperature following equations (3) to (5).

### CH_4_ production and CH_4_ oxidation potentials (in-stream incubations)

Sediment samples (roughly the top 2 cm) were collected by hand using a pre-autoclaved and ethanol-cleaned scoop from three locations in each stream (as technical replicates). Due to the remote locations, repeat autoclaving was not possible, and scoops were reused after cleaning with ethanol and flaming only. Sediment samples were used to fill gas-tight glass vials (three replicates with five vials each, plus two control vials containing only water for CH_4_ production and CH_4_ oxidation, therefore adding up to 34 vials per stream) to one-third-full, along with one-third stream water (3 ml for Iceland, Alaska and Greenland; 12 ml for Svalbard and Kamchatka), for both the CH_4_ production and CH_4_ oxidation incubations, with additional vials receiving only stream water, serving as controls. Vials used to measure methanogenesis were deoxygenated by flushing the headspace and overlying water with oxygen-free nitrogen (grade 5.0, Linde Gas) gas for three minutes each. Vials used to measure potential rates of CH_4_ oxidation received a headspace concentration of 100 ppm ^13^C-CH_4_ (99% ^13^C labelling, Cambridge Isotopes), which was distributed through the vials by vigorous shaking for 30 seconds. Additional vials were filled with sediment and water as above to determine the natural abundance of ^13^C-CO_2_ (that is, ^45^CO_2_) to calculate excess production of ^13^C-CO_2_ from ^13^C-CH_4_. All replicate vials for all time points were placed back into each respective stream to incubate and then sacrificed at four subsequent time points during the next 48 hours by injecting 15 μl of 37% formaldehyde and shaking the vials vigorously for 30 seconds.

Subsequently, CH_4_ production potentials (nmol g^−1^ h^−1^) and CH_4_-to-CO_2_ production ratios were determined from the accumulation of CH_4_ and CO_2_ in the headspace over time using a gas chromatograph fitted with a flame ionization detector as described above. CH_4_ oxidation potentials (nmol g^−1^ h^−1^) were measured by determining the production of ^13^C-CO_2_ in the headspace over time using continuous-flow isotope ratio mass spectrometry^60^. Total ^13^C-CO_2_ production in both the headspace and overlying water was subsequently calculated from stream water pH:

7 $${P}_{\mathrm{tot\_}13\mathrm{DIC}}={P}_{\mathrm{hs\_}13\mathrm{CO}2}\times \left(\,{f}_{\frac{\mathrm{HCO}3}{\mathrm{CO}2}}+1\right)$$

8 $${f}_{\frac{{\rm{HCO}}3}{{\rm{CO}}2}}=\frac{4.15\times {10}^{-7}}{{10}^{-{\rm{pH}}}}$$

where *P*_tot_13DIC_ is the total ^13^C-CO_2_ production and *P*_hs_13CO2_ is the ^13^C-CO_2_ production in the headspace. ${f}_{\frac{{\rm{HCO}}3}{{\rm{CO}}2}}$ is the ratio of hydrogen bicarbonate to dissolved CO_2_ gas calculated from pH according to equation (8).

After analyses of all potential rates, surface water was discarded, and the sediments were dried for three days at 70 °C to determine sediment DW, which was then used to normalize all potential rates to per g DW.

### Normalization of CH_4_ oxidation potentials to a constant initial CH_4_ concentration

In the field we aimed to spike each sediment sample with the same initial concentration of CH_4_, but due to unknown differences in sediment porewater CH_4_ (in the field), there were unavoidable differences in the initial CH_4_ concentrations that came to light on analysis back in the laboratory. Therefore, to isolate the temperature sensitivity of CH_4_ oxidation from any effect of initial CH_4_ concentrations, we normalized the CH_4_ oxidation potentials prior to fitting mixed-effect models using:

9 $${R}_{{\rm{MO}}({\rm{nor}})}={R}_{{\rm{MO}}}\times \frac{{R}_{{\rm{MO}}(\max )}\times {C}_{{\rm{CH}}4({\rm{median}})}}{{k}_{{\rm{m}}}+{C}_{{\rm{CH}}4({\rm{median}})}}\div\frac{{R}_{{\rm{MO}}(\max )}\times {C}_{{\rm{CH}}4({\rm{T}}0)}}{{k}_{{\rm{m}}}+{C}_{{\rm{CH}}4({\rm{T}}0)}}$$

where *R*_MO_ is the raw CH_4_ oxidation potential (nmol g^−1^ h^−1^) calculated in the previous section. *R*_MO(max)_ and *k*_m_ are the maximum rate (586 nmol CH_4_ g^−1^ h^−1^) and the Michaelis constant (3.7 µmol l^−1^), respectively, derived from our previously published empirical Michaelis–Menten kinetic relationship^32,42^. *C*_CH4(T0)_ is the initial concentration of CH_4_ in each sample, and *C*_CH4(median)_ is the median value of 700 nM. *R*_MO(nor)_ is therefore the rate of CH_4_ oxidation normalized to the same median initial CH_4_ concentration.

### Process-level oxidation efficiency of streambed CH_4_

Process-level oxidation efficiency (equation (10)) was calculated as the fraction of porewater CH_4_ oxidized—that is, turnover per hour:

10 $${\rm{Process}}{\textstyle \mathrm{-}}{\rm{level}}\,{\rm{oxidation}}\,{\rm{efficiency}}\,({{\rm{h}}}^{-1})=\frac{{R}_{{\rm{MO}}({\rm{nor}})}}{{C}_{{\rm{pw}}{\rm{\_}}{\rm{CH}}4({\rm{median}})}}$$

where *R*_MO(nor)_ is the normalized CH_4_ oxidation potential (above, nmol g^−1^ h^−1^) and *C*_pw_CH4(median)_ is the median value of CH_4_ concentrations in porewater measured at 2, 4, 6, 8 and 10 cm depth in each stream (nmol g^−1^; see ‘Stream characteristics’; normalized to the DWs in the incubation vials). Note that the CH_4_ oxidation potentials were normalized to the same initial CH_4_ concentration of 700 nM (equation (9)), which was greater than the typical CH_4_ concentrations in the streambed porewater (140 to 560 nM for the first and third quartiles, respectively), and, as a result, oxidation efficiencies (turnover rates) could be greater than 1 h^−1^.

### System-level CH_4_ filter efficiency

The system-level CH_4_ filter efficiency was calculated as 1 minus the ratio of CH_4_ concentration in stream water to that in porewater—that is, the fraction (%) of streambed CH_4_ oxidized before reaching the water column:

11 $${\mathrm{System}}\mathrm{-}{\mathrm{level}}\,{\mathrm{CH}}_{4}\,{\mathrm{filter}}\,{\mathrm{efficiency}}\,({\%})=\left(1-\frac{{C}_{{\mathrm{stream}}\_{\rm{CH}}4}}{{C}_{{\mathrm{pw}}\_{\rm{CH}}4({\mathrm{median}})}}\right)\times 100$$

where *C*_stream_CH4_ is the CH_4_ concentration (nM) in the overlying stream water and is the same as *C*_measured_ in equation (1), and *C*_pw_CH4(median)_ is the same as in equation (10) but here expressed in the original porewater concentration units (nM). Note, *C*_stream_CH4_ would be net of any water column CH_4_ oxidation, which can be significant in turbid clay- or sand-based streams but was not the case here^42^. We and others have shown previously that the streambed sediments in small headwater streams like those reported here dominate the supply of CH_4_ to the overlying stream water^42,43^, and our ratio measure of the system-level CH_4_ filter efficiency provides a comparative measure across cold versus warm streams with similar hydrophysical characteristics (Supplementary Tables 3 and 4).

### Sediment sampling for molecular microbial analysis

Sediment was sampled from roughly the top 2 cm of the streambeds and homogenized in a sterile trough (StarLab) using pre-autoclaved disposable spatulas (VWR). A sub-sample was transferred into pre-labelled 2-ml cryogenic vials (StarLab), transported from the field in a cool box and immediately transferred to a −20 °C freezer (Dometic CFX-65 60 l Portable Compressor Fridge Freezer)^61^. Once back at the University of Essex laboratories, the samples were transferred to −80 °C for long term storage and processing as described below.

### DNA extraction from stream sediment

DNA was extracted from 0.25 g wet sediment using the DNeasy PowerSoil Kit (Qiagen) following the manufacturer’s instructions. For quantification (quantitative PCR (qPCR)) of methanogenic and methanotrophic abundance (gene copy numbers), sediment samples from three locations along each stream transect were assayed, paired with the in situ and potential stream biogeochemical process measurements. DNA metabarcoding of methanogen and methanotroph communities was conducted across a broader stretch of stream transects. The average number of sediment samples per stream was 6 (±1.0).

### Quantification of methanogenic (*mcrA*) and methanotrophic (*pmoA*) copy numbers as a measure of abundance (qPCR)

Two separate qPCR assays were performed for *mcrA* and *pmoA* to determine copy numbers (an estimation of abundance; Supplementary Table 9) in the original sediment samples. qPCR DNA standards were created from end-point PCR amplification where the template DNA was 1 µl of extracted DNA pooled from each environmental sample. The resulting amplicons were purified using a QIAquick PCR purification kit (Qiagen) and quantified using the Invitrogen Quant-iT PicoGreen dsDNA assay kit (Fisher Scientific). Each target gene was assayed separately using a CFX384 Real-Time PCR Detection System (BIO-RAD). Assays were run on 384-well plates (BIO-RAD) and included a serial dilution of the purified standard ranging from 10^−1^ to 10^−9^, non-template (negative) controls and samples, all of which were added in triplicate. qPCR reactions were performed in a 10-µl reaction volume with 1 µl of sample DNA, 5 µl of SensiFAST Sybr Green (Bioline, Reagents Ltd), 0.2 µl of each primer (10 µM) and 3.6 µl of Invitrogen UltraPure DNase/RNase-Free Distilled Water (ThermoFisher Scientific). The primers used to amplify each target gene are presented in Supplementary Table 9. The qPCR conditions to amplify regions of both the *mcrA* and *pmoA* genes were as follows: for *mcrA*, 95 °C for 3 min, followed by 40 cycles at 95 °C for 15 s and 65 °C for 30 s; for *pmoA*, 95 °C for 3 min, followed by 40 cycles at 95 °C for 15 s and 60 °C for 30 s. Melt curve generation was added to the end of each assay for one cycle at 95 °C for 5 s, 65 °C for 5 s and 95 °C for 5 s. An additional 0.25 g of sediment was taken from each of the original samples used for qPCR assays, weighed into a clean weighing boat and dried for 48 hours at 60 °C to determine sediment DWs. Dry mass data were used to express copy numbers of *mcrA* and *pmoA* genes from each sample per g of DW sediment. To account for the recognized multiple copies of *pmoA* in some methanotrophs, we searched KEGG, the genome database and individual published species and found an overall average copy number of 1.5 (see Supplementary Table 9 and the discussion therein), which we used to correct methanotroph abundance (see equation (21) and the related text). As per our earlier publication^15^ and work by Steinberg and Regan^62^, there was no evidence for multiple copies of *mcrA*, and therefore no correction was applied to the methanogen qPCR abundance.

### DNA metabarcoding of methanogen (*mcrA*) and methanotroph (*pmoA*) communities

Amplicon library preparation followed the protocol outlined by Illumina^63^ with PCR conditions optimized for the two target genes. First-stage PCR reactions were performed in a 25-µl reaction volume with 2 µl of DNA template, 12.5 µl appTAQ RedMix (2×) polymerase (Appleton Woods Ltd), 8.5 µl of Invitrogen UltraPure DNase/RNase-Free Distilled Water (ThermoFisher Scientific) and 1 µl of each primer (4 µM), which contained Illumina overhang adaptors and had the same locus-specific sequences as those used for qPCR (Supplementary Table 9). PCR was run in 96-well plates (StarLab) on a 96 Well Thermo Cycler (Applied Biosystems), with three negative controls included per plate. The PCR conditions to amplify each target gene were as follows: 95 °C for 3 min; 35 cycles at 95 °C for 15 s, 55 °C (*mcrA*) or 53 °C (*pmoA*) for 30 s and 72 °C for 30 s; 72 °C for 7 min. Every sample for both genes was then checked for positive amplification by loading into pre-cast Invitrogen 96 Agarose E-gels (2% agarose) (FisherScientific) and run for 13 minutes on an Invitrogen Mother E-Base (FisherScientific). PCR amplicons from the first-stage PCR reactions were cleaned and amplicon libraries were indexed following the Illumina protocol using the Nextera XT Library Prep Kit (Illumina). Indexed amplicon libraries were then cleaned^63^. For each amplicon library, cleaned and indexed individual samples were then quantified using the Invitrogen Quant-It PicoGreen dsDNA assay kit (ThermoFisher Scientific) before the samples were pooled in equimolar concentrations. Final amplicon library concentration was then determined using a NEBNEXT Library Quant Kit for Illumina, and quality checked using an Agilent 2100 Bioanalyzer System (Agilent Technologies). The samples were sequenced on an Illumina MiSeq (600 cycles; reagent kit v3) via the NERC Biomolecular Analysis Facility at the Centre for Genome Research (Liverpool, UK).

### Bioinformatic processing of microbial metabarcoding data

The raw *mcrA* and *pmoA* metabarcoding reads were subjected to quality control, including sequencing trimming, error correction and the removal of poor-quality sequences and chimeric PCR artefacts^64^. Sequences were clustered into species-level operational taxonomic units (OTUs) using VSEARCH^65^ at similarity cut-offs that represent sequence divergence among methanogen and methanotroph species, respectively (85% and 90% similarity for *mcrA* and *pmoA*^66,67^). To correct for frameshift errors and to remove any non-locus-specific OTUs, we used FrameBot with the default settings^68^. To specify the methanogenic pathways or the methanotrophic types associated with each OTU, we placed the OTUs into maximum-likelihood trees constructed from the reference sequences of known methanogen or methanotroph cultures (see Extended Data Figs. 6 and 7 for the phylogenetic trees of *mcrA* and *pmoA* OTUs, respectively). The OTUs were aligned and incorporated into the clades of reference species on the basis of robust phylogenetic trees and subsequently assigned methanogenic pathways or methanotrophic types accordingly (Extended Data Figs. 6 and 7). The trees were constructed from aligned sequences by MUSCLE using a GTR + G + I model in Mega-X (version 10.2.2). After bioinformatic processing, a total of 93 *mcrA* (methanogen) OTUs from 3,793,456 reads and 260 *pmoA* (methanotroph) OTUs from 2,146,501 reads were used for subsequent analysis.

As genus is the highest taxonomic resolution for assigning methanogenic pathways to each OTU (Extended Data Fig. 6), the *mcrA* data were agglomerated at this level and rarefied to 1,000 reads per sample as the method of normalization^69,70^ using the R package phyloseq (version 1.48.0)^71^. As assigning methanotrophic type to *pmoA* OTU only needs taxonomic information at the class level, the *pmoA* data were agglomerated at the class level and then rarefied to the 1,006 reads.

### Statistical analyses

All statistical analyses were performed in R (version 4.2.3)^72^, and all graphical presentations were created using the ggplot2 (version 4.0.0), rnaturalearth (version 1.1.0), rnaturalearthdata (version 1.0.0), sf (version 1.0-21) and leaflet (version 2.2.3) packages^73–77^.

#### Temperature sensitivity of in situ CH_4_ emission, CH_4_ production potential, CH_4_:CO_2_ production ratio and CH_4_ oxidation potential

First, we used principal components analysis^78^ to demonstrate that the design of our natural warming experiment had identified temperature as a key environmental factor as demonstrated previously^49–51^ (Extended Data Figs. 1 and 2). We quantified the temperature sensitivity of in situ CH_4_ emissions (mmol m^−2^ d^−1^), CH_4_ production potential (nmol g DW^−1^ h^−1^), the production ratio CH_4_:CO_2_ (unitless) and CH_4_ oxidation potential (nmol g DW^−1^ h^−1^) using hierarchical Boltzmann–Arrhenius models fit in a fully Bayesian framework. Following Yvon-Durocher et al.^10^, rates were analysed on the natural-log scale as linear functions of a centred inverse-temperature covariate. For an observation *y*_*ijk*_ made in stream *k* in region *j* at absolute temperature *T*_*ijk*_ (K), we defined the centred inverse-temperature predictor as (units: eV^−1^)

12 $${x}_{{ijk}}\equiv \frac{1}{k{T}_{{\rm{c}}}}-\frac{1}{k{T}_{{ijk}}}$$

where *k* = 8.62 × 10^−5 ^eV K^−1^. The centring temperature, *T*_c_, was set to the dataset-specific mid-temperature to improve parameter interpretability (*T*_c_ = 18 °C for emission and 14 °C for production, the ratio of CH_4_ to CO_2_ production and oxidation). Because the model is linear in *x*, the slope parameter is directly interpretable as an activation energy (temperature sensitivity) in eV, and the intercept gives the (log) rate at *T* = *T*_c_ (‘capacity’). See Extended Data Fig. 4 for variation across regions in the posterior distributions of temperature sensitivities.

Let *y*_*ijk*_ = ln(CH_4_ emission). We modelled

13 $${y}_{{ijk}}{\mathscr{ \sim }}{\mathscr{N}}\left({\mu }_{{ijk}},\,\sigma \right)$$

14 $${\mu }_{{ijk}}=\,\alpha +\beta {x}_{{ijk}}+{a}_{0j}+{b}_{0{jk}}+\left({a}_{1j}+{b}_{1{jk}}\right){x}_{{ijk}}$$

where *α* is the intercept (that is, the capacity at *T*_c_), *β* is the slope (that is, the activation energy, $\overline{{E}_{\mathrm{ME}}}$ in eV), *a*_0*j*_ is the region-specific intercept shift (that is, how region *j* differs in log-capacity at *T*_c_), *a*_1*j*_ is the region-specific slope shift (that is, how region *j*’s activation energy differs from *β*), *b*_0*jk*_ is the stream-specific intercept shift within region *j* and *b*_1*jk*_ is the stream-specific slope shift within region *j*. Here stream effects are nested in regions. Random intercepts and slopes were allowed at both the region and stream levels and were correlated within level:

15 $$\begin{array}{l}{a}_{0j}\\ {a}_{1j}\end{array} \sim {\mathscr{N}}\left(0,\,\mathop{\sum }\limits_{\mathrm{Region}}\right),\,\begin{array}{l}{b}_{0{jk}}\\ {b}_{1{jk}}\end{array} \sim {\mathscr{N}}\left(0,\,\mathop{\sum }\limits_{\mathrm{Stream}}\right)$$

Priors $\alpha \sim {\mathscr{N}}\left(0,\,2\right)$ and $\beta \sim {\mathscr{N}}\left(0.6,\,0.3\right)$ were weakly informative around canonical metabolic sensitivities^79^. Standard deviations of all random-effect terms were given as ~ Exponential(5). Correlation matrices ∑ were assigned LKJ(*η* = 2) priors, and residuals were given as *σ* ~ *t*_*v*=3_(0, 2.5). This model corresponds to the brms formula ln_flux ~ 1 + cent_Abs + (1 + cent_Abs | Region/Stream), with family = gaussian() and the priors as described above.

#### (ii) CH_4_ production potentials

##### CH_4_ production potentials

For *y*_*ijk*_ = ln(CH_4_ production), we used the same structure as for emissions to allow both capacity and temperature sensitivity to vary at the region and stream levels:

16 $${y}_{{ijk}}={\mathscr{N}}{\mathscr{(}}\alpha +\beta {x}_{{ijk}}+{a}_{0j}+{b}_{0{jk}}+\left({a}_{1j}+{b}_{1{jk}}\right){x}_{{ijk}},\,\sigma )$$

Parameters and priors are as described in equations (14) and (15) except that the prior on the slope was broader, $\beta \sim {\mathscr{N}}\left(0.6,\,1\right)$, reflecting greater uncertainty around the canonical metabolic sensitivities in production^10^. The slope *β* describes the activation energy for CH_4_ production potentials (that is, $\overline{{E}_{\mathrm{MP}}}$ in eV). This model corresponds to the brms formula ln(CH_4_ production) ~ 1 + cent_Abs + (1 + cent_Abs | Region/Stream), with family = gaussian().

##### CH_4_:CO_2_ production ratio

For *y*_*ijk*_ = ln(CH_4_:CO_2_ production ratio), we fit the same hierarchical structure as for emissions and production (see equations (13) and (14) and the related description of the parameters; note that the slope *β* describes the activation energy for the CH_4_:CO_2_ production ratio—that is, $\bar{{E}_{{\rm{MR}}}}$ in eV). The priors are as described above (equation (15)) except a wider prior on the slope, $\beta \sim {\mathscr{N}}\left(0.6,\,1\right)$, reflecting weaker prior information on temperature sensitivity for the ratio. The corresponding brms formula was ln(CH_4_:CO_2_ production ratio) ~ 1 + cent_Abs + (1 + cent_Abs | Region/Stream), with family = gaussian().

##### CH_4_ oxidation potentials

For *y*_*ijk*_ = ln(CH_4_ oxidation), to avoid over-parameterization, we allowed random intercepts at both the region and stream levels and random slopes only at the region level, and we constrained region-level intercept–slope correlations to zero:

17 $${y}_{{ijk}}={\mathscr{N}}{\mathscr{(}}\alpha +\beta {x}_{{ijk}}+{a}_{0j}+{b}_{0{jk}}+{a}_{1j}{x}_{{ijk}},\,\sigma )$$

18 $${a}_{0j} \sim {\mathscr{N}}(0,\,{\tau }_{\alpha ,\mathrm{Region}}^{2}),\,{a}_{1j} \sim {\mathscr{N}}(0,\,{\tau }_{\beta ,\mathrm{Region}}^{2}),\,{b}_{0{jk}} \sim {\mathscr{N}}(0,\,{\tau }_{\alpha ,\mathrm{Stream}}^{2})$$

where ${\tau }_{\alpha ,{\rm{Region}}}^{2}$ is the variance of the region-level intercepts, ${\tau }_{\beta ,{\rm{Region}}}^{2}$ is the variance of the region-level slopes and ${\tau }_{\alpha ,{\rm{Stream}}}^{2}$ is the variance of the stream-level intercepts. The slope *β* describes the activation energy for CH_4_ oxidation potentials—that is, $\overline{{E}_{\mathrm{MO}}}$ in eV. The remaining parameters are as described in equation (14). Priors for intercept and slope were as before, $\alpha \sim {\mathscr{N}}\left(0,\,2\right)$ and $\beta \sim {\mathscr{N}}\left(0.6,\,0.3\right)$, reflecting canonical metabolic sensitivities^79^. Standard deviations of all random-effect terms were given as ~ Exponential(5). Correlation matrices ∑ were assigned LKJ(*η* = 2) priors, and residuals were given as *σ* ~ *t*_*v*=3_(0, 2.5).

This model corresponds to the brms formula ln(CH_4_ oxidation) ~ 1 + cent_Abs + (1 | Region) + (0 + cent_Abs || Region) + (1 | Region:Stream), with family = gaussian() and the priors as described above.

#### Temperature sensitivity of methanogen and methanotroph abundance (*mcrA* and *pmoA* copy numbers via qPCR)

There were 9 and 16 zeros in the methanogen and methanotroph abundance data, respectively. As the concentration of total DNA extracted from the sediments of these high-latitude, pristine streams was low (median of 0.7 ng μl^−1^; see Supplementary Table 6 for examples), zero copy numbers of functional genes were supported and therefore retained in the dataset. To address the zero copy numbers, a hierarchical Bayesian regression framework using a zero-inflated negative binomial distribution^80^ was applied:

19 $${y}_{{ijk}}={\rm{Zero}}{\textstyle \mathrm{-}}{\rm{inflated}}\,{\rm{negative}}\,{\rm{binomial}}({\mu }_{{ijk}},\,\sigma )$$

where *y*_*ijk*_ = *mcrA* abundance or *pmoA* abundance (copy per g DW) in sample *i* from stream *k* nested in region *j*.

For *mcrA* abundance, the same hierarchical structure as previously applied for emissions and production was applied (Abundance ~ 1 + cent_Abs + (1 + cent_Abs | Region/Stream); see equations (13) and (14) and the corresponding parameter definitions (note that the slope *β* describes the activation energy for methanogen abundance—that is, $\overline{{E}_{\mathrm{Mab}}}$ in eV). Weakly informative priors $\alpha \sim {\mathscr{N}}\left(2,\,4\right)$ and $\beta \sim {\mathscr{N}}\left(0.6,\,0.3\right)$ were performed to reflect greater uncertainty in abundance scaling. Standard deviations of all random-effect terms were given as ~ Exponential(5). Correlation matrices ∑ were assigned LKJ(*η* = 2). Residual variation was implicitly modelled within the zero-inflated negative binomial family and therefore not specified.

For *pmoA* abundance, to avoid over-parameterization, a hierarchical structure with uncorrelated varying slopes was used, the same as for oxidation (Abundance ~ 1 + cent_Abs + (1 | Region) + (0 + cent_Abs || Region) + (1 | Region:Stream); see equations (17) and (18), and note that the slope *β* describes the activation energy for methanotroph abundance—that is, $\overline{{E}_{\mathrm{MOab}}}$ in eV). Therefore, no correlation parameters or LKJ priors on correlation matrices were specified. Priors on fixed effects and random-effect standard deviations were performed the same as for *mcrA* abundance.

As integer counts are required to fit a Bayesian regression framework with a zero-inflated negative binomial distribution, we fitted the correction coefficient of 1.5, which accounted for multiple copies of *pmoA*, as an offset to avoid the production of fractions (see Supplementary Tables 5 and 9 for model fitting and the rationale for the correction coefficient, respectively; and see Extended Data Fig. 4 for variation across regions in the posterior distributions of temperature sensitivities).

#### Changes of community diversity along the natural warming gradient

The methanogen or methanotroph communities were first assessed in terms of Shannon index estimated for each sample using the estimate_richness function from the phyloseq package^71^. For *y*_*ijk*_ = Shannon indexes, we fit the same hierarchical structure as for emissions and production (see equations (13) and (14) and the related descriptions of the parameters). The model corresponds to the brms formula Shannon ~ 1 + cent_Abs + (1 + cent_Abs | Region/Stream), with family = gaussian(). The priors are as described above (equation (15)) except with a wider slope prior to reflect weaker a priori constraints on the ratio, $\beta \sim {\mathscr{N}}\left(0,\,1\right)$.

#### Community compositional changes along the natural warming gradient

Prior to analysing changes in community composition, the hydrogenotrophic and acetoclastic *mcrA* genera, assigned to each OTU as per their phylogeny, were grouped at the family level, while the methylotrophic genera were all grouped together (Extended Data Figs. 6 and 7 and related discussion). For example, *Methanobacterium* and *Methanothermobacter* were grouped into Methanobacteriaceae, while *Methanosphaerula*, *Methanolinea* and *Methanoregula* were grouped as Methanoregulaceae. As *Methanocella* and *Methanothrix* were the only genera detected in the streambed sediments in Methanocellaceae and Methanotrichaceae, respectively, these two genera were not grouped but were represented using their family names for consistency. Our grouping strategy represented a trade-off between degrees of freedom for analysis and retaining enough resolution of the composition of the major methanogen groups—that is, acetoclasts and hydrogenotrophs.

Unlike *mcrA* sequences, the *pmoA* sequences were already agglomerated at the class level to separate the two types of methanotrophs (Extended Data Fig. 7); therefore, no further taxonomic integration was needed.

Furthermore, to improve the normality of the data, the relative abundances of each category (Rab) were arcsine-transformed using:

20 $${\rm{Tab}}={{\rm{arcsine}}({\rm{Rab}}}^{0.5})$$

where Tab is the transformed relative abundance.

We analysed *y*_*ijk*_ = Tab (that is, the relative abundance of a taxonomic category for sample *i* in stream *k* within region *j*) using hierarchical Bayesian linear mixed models including an interaction between temperature and category:

21 $${y}_{{ijk}}={\mathscr{N}}{\mathscr{(}}\alpha +{\beta }_{1}{x}_{{ijk}}+\mathop{\sum }\limits_{g=1}^{G}{\gamma }_{g}{G}_{g,{ijk}}+\mathop{\sum }\limits_{g=1}^{G}{\delta }_{g}{x}_{{ijk}}\times {G}_{g,{ijk}}+{a}_{0j}+{b}_{0{jk}},\,\sigma )$$

22 $${a}_{0j} \sim {\mathscr{N}}(0,\,{\tau }_{\alpha ,\mathrm{Region}}^{2}),\,{b}_{0{jk}} \sim {\mathscr{N}}(0,\,{\tau }_{\alpha ,\mathrm{Stream}}^{2})$$

where *α* is the intercept, *x*_*ijk*_ is the inverse-temperature predictor (equation (12)), *G*_*g*,*ijk*_ is indicator variables for each taxonomic category *g* with *G* levels (*G* = 5 and 2 for the methanogen and methanotroph communities, respectively), *β*_1_ is the main effect of temperature (for the baseline *g* = 1—that is, Methanocellaceae for the methanogen community and type I for the methanotroph community), *δ*_*g*_ is the category-specific temperature interactions and ${\beta }_{1}+{\sum }_{g=1}^{G}{\delta }_{g}$ is therefore the overall slope for taxonomic category *g* (denoted as *β* in Fig. 3 for clarity). *γ*_*g*_ is the category-specific intercept relative to baseline *g* = 1 (that is, Methanocellaceae for the methanogen community and type I for the methanotroph community). *a*_0*j*_ and *b*_0*jk*_ are the region- and stream-specific intercepts, and here the stream effects are nested in regions. To avoid over-parameterization, no random slopes were included.

We assigned weakly informative priors, $\alpha \sim {\mathscr{N}}\left(0,\,2\right)$ and ${\beta }_{1}{\mathscr{ \sim }}{\mathscr{N}}\left(0,\,0.5\right)$, reflecting reasonable constraints on the parameters. Standard deviations of all random-effect terms were given as ~ Exponential(2), and residuals were given as *σ* ~ *t*_*v*=3_(0, 2.5). The corresponding brms formula is Tab ~ 1 + cent_Abs × Category + (1| Region/Stream), with family = gaussian().

#### Analysis of process-level CH_4_ oxidation efficiency and system-level CH_4_ filter efficiency

We analysed the process-level CH_4_ oxidation efficiency (log-transformed) and system-level CH_4_ filter efficiency using hierarchical Bayesian linear mixed models. To evaluate the changes along the natural warming gradient, the samples were grouped as streams above or below the median stream temperature of 10.5 °C as either ‘warm’ or ‘cold’, respectively, and fitted as a binary factor (CW_*ijk*_). For *y*_*ijk*_ = ln(Eff._MO)—that is, the process-level CH_4_ oxidation efficiency for sample *i* from stream *k* in region *j*—a model with Gaussian regression was fitted as:

23 $${y}_{{ijk}}={\mathscr{N}}(\alpha +\beta \times {{\rm{CW}}}_{{ijk}}+{a}_{0j}+{b}_{0{jk}}+{a}_{1j}{x}_{{ijk}}+{b}_{1{jk}}\times {{\rm{CW}}}_{{ijk}},\,\sigma )$$

where *α* is the intercept representing the process-level CH_4_ oxidation efficiency for cold streams and *β* the effect of warm streams relative to the cold. *a*_0*j*_ and *a*_1*j*_ are the region-specific intercept and slope. *b*_0*jk*_ and *b*_1*jk*_ are the stream-specific intercept and slope nested in region. Random intercepts and slopes were allowed at both the region and stream levels and were correlated within level (equation (15)).

As *y*_*ijk*_ = Eff._filter—that is, the system-level CH_4_ filter efficiency—is a continuous proportion bounded between 0 and 1, a model using beta regression with logit link was fitted:

24 $${y}_{{ijk}}={\rm{beta}}(\alpha +\beta \times {{\rm{CW}}}_{{ijk}}+{a}_{0j}+{b}_{0{jk}}+{a}_{1j}{x}_{{ijk}}+{b}_{1{jk}}\times {{\rm{CW}}}_{{ijk}},\,\varphi )$$

where the parameters are analogous to those described above, and *φ* is the beta distribution parameter.

Weakly informative priors, $\alpha \sim {\mathscr{N}}\left(0,\,2\right)$ and ${\beta }_{1}{\mathscr{ \sim }}{\mathscr{N}}\left(0,\,1\right)$, were performed to regularize inference. Standard deviations of all random-effect terms were given as ~ Exponential(5). Correlation matrices ∑ were assigned LKJ(*η* = 2) priors. Residuals were given as *σ* ~ *t*_*v*=3_(0, 2.5) (in the Gaussian model) and precision *φ* ~ gamma(2, 0.1) (in the beta model). The corresponding brms formulas are ln(Eff._MO) ~ 1 + cold_warm + (1 + cold_warm | Region/Stream), with family = gaussian(), and Eff._filter ~ 1 + cold_warm + (1 + cold_warm | Region/Stream), with family = Beta().

#### Estimation, convergence and uncertainty reporting

All models were fitted with 4 chains × 4,000 iterations (2,000 warm-up, 2,000 saved), seed = 1,234, using brms (which uses Stan’s NUTS sampler) with adapt_delta = 0.99 and max_treedepth = 15 for robust convergence. Weakly informative priors incorporated established expectations for metabolic temperature dependences while remaining conservative.

Convergence was assessed by monitoring $\hat{R}$ (all targeted <1.01), effective sample sizes and absence of divergent transitions (achieved via adapt_delta = 0.99). For each parameter, we report posterior medians and equal-tailed 95% CIs (Supplementary Tables 5, 7 and 8). Region-specific activation energies *E*_*j*_ were obtained as *β* + *a*_1*j*_ and, for models with stream-level slopes, *β* + *a*_1*j*_ + *b*_1*jk*_ when stream-level sensitivities were required.

##### Model checking and out-of-sample performance

We evaluated predictive fit using Pareto-smoothed importance-sampling leave-one-out cross-validation (PSIS-LOO) with moment matching and automatic refitting of problematic observations (loo(…, moment_match = TRUE, reloo = TRUE)). We inspected Pareto-*k* diagnostics (target *k* < 0.7) and compared LOO-IC across alternative structures during model development. Posterior predictive checks included distributional overlays, group-wise violin PPCs by region, LOO-PIT Q–Q plots and average error versus predictor plots to detect mis-specification (all implemented via bayesplot/loo).

##### Derived quantities and visualization

To visualize overall (fixed-effect) temperature responses, we plotted draws of the expected mean response with random effects set to zero (re_formula = NA), along with 95% credible ribbons. For region-level curves, we included region random effects (re_formula = ~(1 + cent_Abs | Region)) and averaged over streams. For comparability across regions, we also displayed capacity-normalized responses by subtracting, from each observation, the posterior mean region-level intercept; this centres panels at their region-specific log capacity at *T*_c_. Region-wise posterior densities of activation energies and capacities were obtained by transforming posterior draws (slopes and intercepts, respectively; capacities are presented on the original scale via exponentiation).

##### Software and data processing

All analyses were performed in R using brms^80^ (version 2.23.0) for model fitting, loo (version 2.8.0) for PSIS-LOO, tidybayes (version 3.0.7) for extracting/transforming posterior draws, posterior (version 1.6.1)/bayesplot (version 1.14.0) for diagnostics and ggplot2 for visualization. Complete brms formulas, priors, sampler settings and plotting code are provided in the accompanying scripts.

#### Predicted exponential increase in CH_4_ production potential, CH_4_:CO_2_ production and methanogen abundance

The increase in CH_4_ production potential, CH_4_:CO_2_ production and methanogen abundance along the natural warming gradient was predicted using their apparent activation energies as:

25 $$\mathrm{prd}(T)={{\rm{e}}}^{E\left(\frac{1}{k{T}_{{\rm{c}}}}-\frac{1}{kT}\right)}-{{\rm{e}}}^{E\left(\frac{1}{k{T}_{{\rm{c}}}}-\frac{1}{k{T}_{0}}\right)}$$

where prd(*T*) is the predicted increase in CH_4_ production, CH_4_:CO_2_ production or methanogen abundance along a temperature gradient from 0 °C to 30 °C (the same as seen in the naturally warmed streams in this study). *E* is the apparent activation energy estimated with the hierarchical Bayesian linear mixed models in equations (16) and (19)—that is, $\overline{{E}_{\mathrm{MR}}}$ = 1.15 eV, $\overline{{E}_{\mathrm{MP}}}$ = 0.73 eV and $\overline{{E}_{\mathrm{Mab}}}$ = 0.41 eV. *k* is the Boltzmann constant at 8.62 × 10^−5 ^eV K^−1^. The term $\frac{1}{k{T}_{{\rm{c}}}}$ was used to centre the plot to a middle temperature of 14 °C as above for estimating activation energy. As we aimed to demonstrate an exponential increase driven by the apparent activation energy, the prediction curves were set to a starting point of 0 by *T*_0_, the lowest value of the temperature gradient—that is, 0 °C.

### Reporting summary

Further information on research design is available in the Nature Portfolio Reporting Summary linked to this article.

## Online content

Any methods, additional references, Nature Portfolio reporting summaries, source data, extended data, supplementary information, acknowledgements, peer review information; details of author contributions and competing interests; and statements of data and code availability are available at 10.1038/s41558-026-02649-2.

## Supplementary information

Supplementary Information Supplementary Figs. 1–4, Tables 1–9, Methods and Discussion.

Reporting Summary

Peer Review File

## Source data

Source Data Figs. 1–4 and Extended Data Figs. 2, 4 and 5 All source data are provided in a single file with clearly labelled tabs. See the ‘Meta_data’ tab for detailed descriptions.

Source Data Extended Data Fig. 6 Phylogenetic tree data for the *mcrA* community.

Source Data Extended Data Fig. 7 Phylogenetic tree data for the *pmoA* community.

## Data Availability

The raw sequencing data for both methanogens and methanotrophs are available via the NCBI SRA online repository under the BioProject numbers PRJNA1119395 for methanogens and PRJNA1119373 for methanotrophs. Source data are provided with this paper.
