# Supplementary material for: A fixed methane filter maximizes freshwater emissions under warming
Source: Nat Clim Chang. 2026 Jun 5;16(6):704–11. doi: 10.1038/s41558-026-02649-2 (PMC13249575; doi:10.1038/s41558-026-02649-2)
Supplement: Supplementary file 1 — Supplementary Figs. 1–4, Tables 1–9, Methods and Discussion. [file 41558_2026_2649_MOESM1_ESM.pdf]

nature climate change

Supplementary information https://doi.org/10.1038/s41558-026-02649-2

A fixed methane filter maximizes freshwater emissions under warming

In the format provided by the authors and unedited

Supplementary Information

Table of contents

Supplementary Methods 2

Supplementary Discussion: Generality of our findings across freshwater systems 3

Supplementary Fig. 4 | The degree of CH_4 over-saturation in stream water increased along the natural warming gradient........ 7

Supplementary Table 1 | Current global CH_4 emissions and estimated increases with 2.5°C of warming under SSP2-4.5 for either a fixed or optimum CH_4 filter scenario. 8

Supplementary Table 2 | Idealised complete mineralisation of glucose to CO_2 and CH_4. 9

Supplementary Table 3 | Hydrophysical characteristics for the streams in the five high-latitude regions. 10

Supplementary Table 4 | Chemical characteristics for the streams in the five high-latitude regions. 11

Supplementary Table 5 | Parameter estimates to characterize the temperature sensitivity in methane-related processes.. 12

Supplementary Table 6 | Subset of sample DNA concentrations for downstream molecular work 15

Supplementary Table 7 | Parameter estimates to evaluate changes in community diversity and community compositional changes along the natural warming gradient.. 16

Supplementary Table 8 | Parameter estimates to evaluate process-level CH_4 oxidation efficiency and system-level CH_4 filter efficiency.. 20

Supplementary Table 9 | Primer sets for qPCR and NGS. 22

References 24

Supplementary Methods

Nutrient analysis for total inorganic nitrogen (TIN: NO_3^-+ NO_2^-+ NH_4^+) and soluble reactive phosphorus (SRP) in stream water

Samples of surface water (7 mL) for nutrient analyses were collected from each stream at three equally spaced locations and filtered in the field using PES Syringes fitted with 0.45μm filters (Gilson, UK). Samples were stored in a freezer (-20 °C) within 8 hours of sampling. Back at the laboratory, concentrations of nitrate (NO_3^-), nitrite (NO_2^-), ammonium (NH_4^+), and soluble reactive phosphorus (SRP) were measured colourimetrically using a segmented flow auto-analyser (Skalar, San^{++}, Breda, The Netherlands), according to Kirkwood (1996)[1]. The limits of detection for NO_2^-, NO_x^- (NO_2^- + NO_3^-), NH_4^+ and SRP are 0.05 μM, 0.1 μM, 0.2 μM and 0.05 μM, respectively[2]. The sum of NO_3^-, NO_2^- and NH_4^+ is presented as total TIN in Supplementary Table 4.

Supplementary Discussion: Generality of our findings across freshwater systems

Emissions

Because our measurements were made at a single point in the growing season, it could be argued that we missed any seasonal variation in the observed temperature – emission relationship. However, high-resolution seasonal wetland time series from Chang et al. (2021)[3] show that although CH_4 flux and temperature exhibit hysteresis throughout the year, re-analysis of their dataset yields an activation energy of 0.40 eV. This value is statistically indistinguishable from the 0.58 eV we derived from our stream network spanning the Northern Hemisphere (Supplementary Fig. 1). Thus, where temporal data exist, they converge on a comparable temperature sensitivity inferred from our spatial gradients. This also agrees with our earlier meta-analysis of 127 globally distributed ecosystems[4], based explicitly on seasonal variation in CH_4 flux and temperature. Together, these independent lines of evidence – one site-level and temporal, the other global and seasonal – demonstrate that the absence of within-stream temporal sampling in our dataset does not compromise the robustness or generality of our findings.

It is also notable that the absolute emission (average rate of emission at the same temperature of 18 °C, the mid-temperature of emission samples of this study, see Methods) in Chang et al.'s wetland (5.3 mmol CH_4 m^{-2} d^{-1}) was ~5.5-fold higher than that from our streams (0.96 mmol CH_4 m^{-2} d^{-1}). Despite these differences in each system's capacity to emit CH_4, their emissions share comparable temperature sensitivities (0.40 vs. 0.58 eV, respectively, with overlapping 95% credible intervals, Supplementary Fig. 1). This reinforces the conclusion that while absolute fluxes differ between systems, the emergent temperature sensitivity is conserved across both time and space.

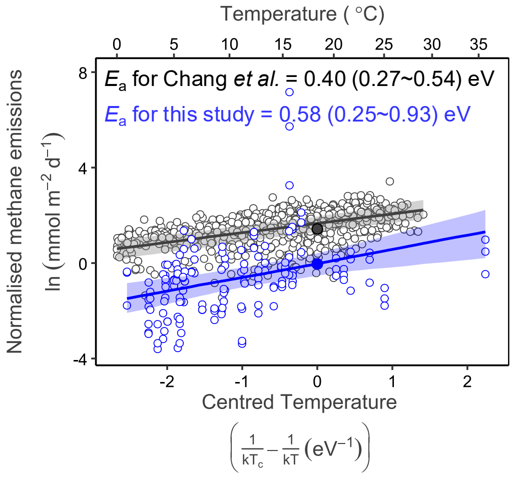

Supplementary Fig. 1 | Temperature dependence of CH_4 emissions across temporal and spatial datasets. Seasonal wetland CH_4 flux data from Chang et al. (2021, blue, n=677 daily measurements in one wetland from 2015 to 2018)[3] compared with our stream data (black, n=148 for 51 independent streams across 5 high latitude regions). Despite hysteresis in the seasonal record, both datasets yield statistically indistinguishable activation energies (0.40 vs. 0.58 eV, shading gives overlapping 95% credible intervals, also in parentheses). While absolute emissions differed (~5.5-fold higher in the wetland at the same 18 °C, filled circles, intercepts at Tc, 0), the emergent temperature sensitivity of emissions was conserved across temporal and spatial scales. The activation energies were quantified using hierarchical Boltzmann–Arrhenius models fit in a fully Bayesian framework as per the figures in the main text, see Methods.

Production

Similarly, because our natural experiment was based in oligotrophic, high-latitude streams with lower CH_4 production potentials than are typical for lakes, wetlands, or tundra soils, it might be expected that the temperature sensitivity may not be representative of higher CH_4 potentials. Yet in our earlier synthesis of riverine sediments, absolute CH_4 production (average rate of production at the same temperature of 14 °C, the same as the mid-temperature for measuring production in this study, see Methods) spanned more than four orders of magnitude as a function of organic matter content, but the temperature sensitivity remained conserved[5] (Supplementary Fig. 2A). Extending this analysis to other ecosystems[6] revealed the same pattern: lakes, wetlands, tundra soils, and rice paddies differ greatly in absolute potential rates but share a common temperature sensitivity (Supplementary Fig. 2B). Importantly, even though the CH_4 production potentials for our new high-latitude stream data were the lowest (blue, 2B), they fall directly on this cross-system relationship, demonstrating that even where absolute rates vary by >10,000-fold, the emergent temperature sensitivity of CH_4 production is robust across ecosystems.

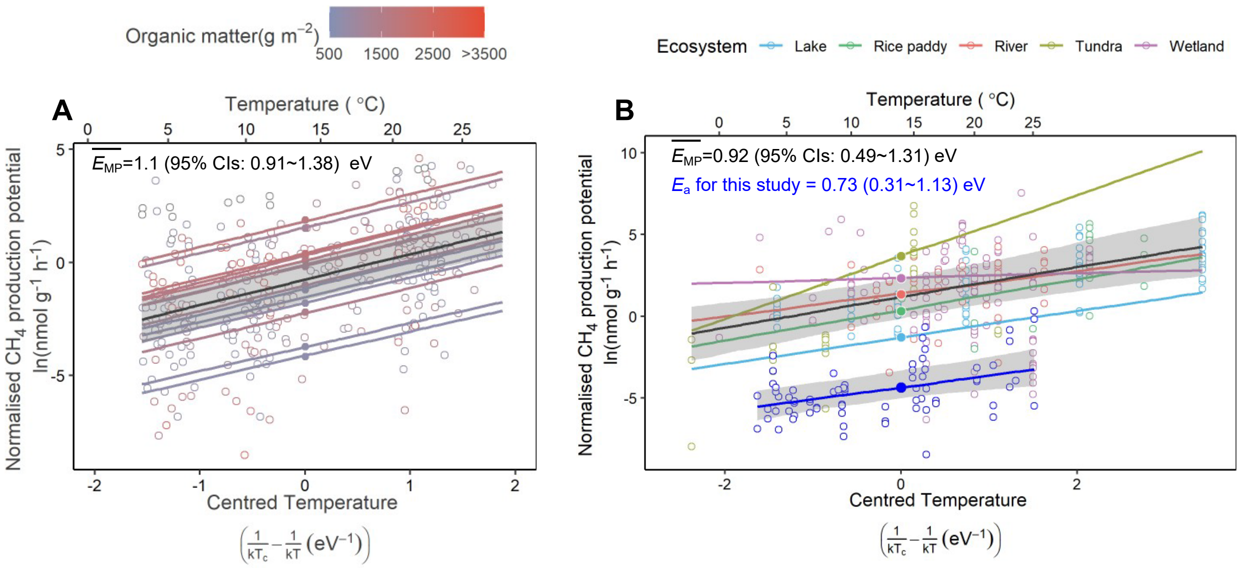

Supplementary Fig. 2 | Temperature sensitivity of CH_4 production across carbon gradients and ecosystem types. A, Stream sediment CH_4 production rates related to sediment organic matter content, showing >10,000-fold variation in absolute production rates (filled circles, intercepts at T_c, 0) but a conserved temperature sensitivity (1.1 eV). **B,** CH_4 production across five freshwater ecosystem types (lakes, rice paddies, rivers, tundra soils, wetlands) also revealed large differences in absolute production rates (filled circles, intercepts at Tc, 0) but a common temperature sensitivity (0.92 eV, shading gives 95% credible intervals, also in parentheses). Our high-latitude stream data (0.73 eV) overlay directly on this cross-system relationship. Data in A (n=371 observations across 14 independent streams) and B (n=336 observations across 5 ecosystem types from 32 studies) were published earlier[5,6] but, for consistency, the activation energies were derived again here using hierarchical Boltzmann–Arrhenius models fitted in a fully Bayesian framework (as per the figures in the main text, and see Methods).

Ebullition

Ebullition has the potential to bypass the CH_4 filter and could therefore undermine the broader applicability of our conclusions. In our high-latitude streams, porewater CH_4 was too low (nM vs. mM typically required) to drive ebullition as we have shown before[7]. In systems where

ebullition is high, such as silty riverbed sediments, ebullition is known to scale with CH_4 concentration, diffusive fluxes and temperature[8]. Our own experimental warming[9] also confirmed this proportionality (Supplementary Fig. 3). Thus, although ebullition may contribute a substantial share of emissions in some settings, the fraction of total CH_4 production oxidised before emission — i.e. system-level filter efficiency — is still expected to be conserved.

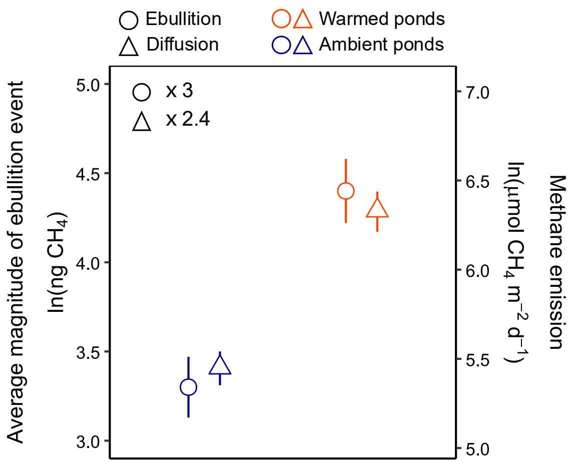

Supplementary Fig. 3 | Proportional response of diffusive and ebullitive CH_4 emissions to experimental warming. Experimental +4 °C warming increased both diffusive (mean ± s.e., *n*=3555 from 7 warmed and 7 ambient ponds) and ebullitive fluxes (mean ± s.e., *n*=198 from 7 warmed and 7 ambient ponds) proportionately, confirming that even when ebullition contributes substantially to total emissions, system-level CH_4 filter efficiency remains fixed. From Zhu et al. (2020)[9].

Oxidation and abundance

A further consideration is whether our conclusions about fixed filter efficiency can be generalised, given that comparable datasets for CH_4 oxidation and methanotroph abundance are scarce. This scarcity is precisely what makes our study novel: across five independent regions spanning the Northern Hemisphere, we consistently found that oxidation potentials increased with warming, but process-level efficiency (i.e., the turnover rate for porewater CH_4 per hour; Eq. 10, Methods) and system-level filter efficiency (i.e., derived from streambed to stream water CH_4 ratio Eq. 11, Methods) remained fixed. Replication of this outcome across distinct regions suggests that the relative responses of production and oxidation are likely to be general features of freshwater CH_4 cycling, even if absolute rates differ. For example, while significant *amounts* of CH_4 can be oxidised in the stratified water column of lakes and the turbid waters of large sluggish rivers and estuaries[10,11] we contend the *relative* response of CH_4 oxidation to CH_4 production would be conserved.

Conclusion

Taken together, these considerations support the generality of our central conclusion: under warming, CH_4 production increases at a rate that CH_4 oxidation can match but not exceed, leaving system-level filter efficiency fixed and emissions rising. While the previous datasets provided here demonstrate conserved temperature sensitivities for emissions and production across ecosystems, our natural experiment shows parallel relationships at multiple levels of organisation — from methanogen and methanotroph abundance, to production and oxidation

potentials, through to emissions. This consistency across scales and across multiple regions spanning the Northern Hemisphere strongly suggests that the fixed filter efficiency we observed in streams in response to warming is likely to be a general property of freshwater CH_4 cycling.

Supplementary Fig. 4 | The degree of CH_4 over-saturation in stream water increased along the natural warming gradient (*n*=148 for 51 streams). The degree of CH_4 saturation is the ratio of the measured CH_4 concentration to the concentration at atmospheric equilibration at *in situ* stream temperature ($C_{[measured]}/C_{[saturation]} \times 100$ %, see equation [1] in the main text). Like streams worldwide that are recognised as sources of CH_4 to the atmosphere[3], all the high-latitude streams here were over-saturated in CH_4 and the overall average degree of over-saturation increased with a temperature sensitivity of 0.53 eV (i.e., activation energy, 95% CI:0.10 to 0.97 eV) along our natural temperature gradient from 1°C to 36°C. Solid line and shaded areas are posterior median and 95% credible intervals, respectively, both extracted from a hierarchical Bayesian linear mixed model.

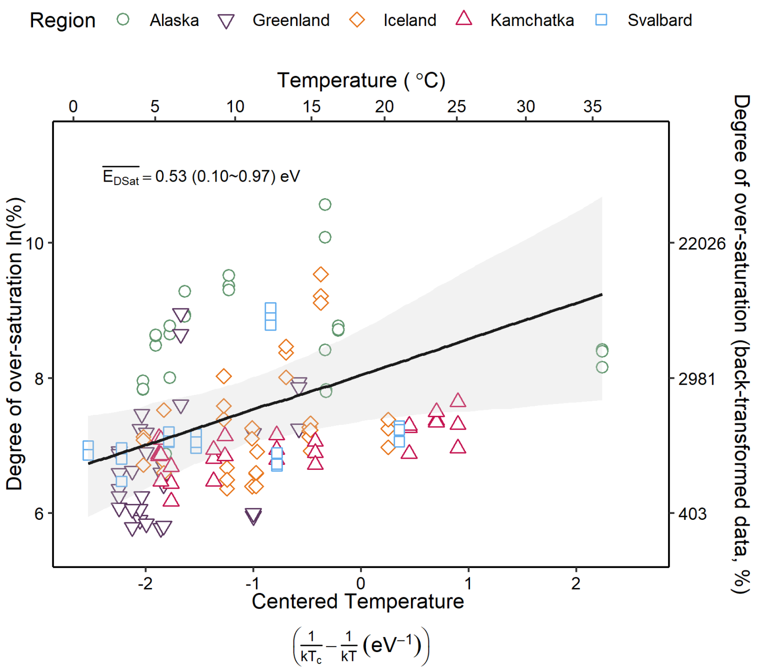

Supplementary Table 1 | Current global CH_4 emissions and estimated increases with 2.5°C of warming under SSP2-4.5 for either a fixed or optimum CH_4 filter scenario.

	Scenarios		
	Current scenario	2.5°C-fixed scenario	2.5°C-optimum scenario
**CH_4 production** (Tg per year)	1437	2003	2003
**CH_4 filter** (%)	70	70	78
**CH_4 emission** (Tg per year)	431	601	431

The CH_4 filter oxidises the majority (typically 70% to 90%) of CH_4 produced in anoxic sediments back to CO_2 before emission to the atmosphere. The current global rate of CH_4 emissions from freshwater ecosystems is estimated to be 431 Tg CH_4 per year[12] and a conservative estimate of total CH_4 production would, accordingly, be 1437 Tg per year (i.e., 431/(100%-70%)=1437, Fig. 1A – current scenario). All 1437 Tg produced per year could potentially be emitted to the atmosphere without the CH_4 filter. Under a future warming scenario of 2.5°C (projected for 2100, SSP2-4.5)[13], and with the recognised temperature sensitivity for methanogenesis of 0.96 eV[4], CH_4 production will increase by 1.4-fold and so will emissions if the CH_4 filter remains fixed at the same 70% efficiency (Fig. 1A – fixed scenario). In comparison, if the efficiency of the CH_4 filter increases disproportionately to 78% i.e., under the optimum scenario, emissions will be constrained at their current level of 431 Tg per year (100%-(431/2003) × 100%=78%) (Fig. 1A – optimum scenario).

Supplementary Table 2 | Idealised complete mineralisation of glucose to equimolar amounts of CO_2 and CH_4 (reaction 4) through a coupling of fermentation (reaction 1) to the hydrogenotrophic (reaction 2) and acetoclastic (reaction 3) pathways of methanogenesis, after[6,14]. Note that the idealised 1:1 ratio for CO_2 to CH_4 production (reaction 4) can only be realised if all of the fermentation products (2 moles of CH_3COOH, 1 CO_2 and 4 moles of H_2) are subsequently used in methanogenesis, which seldom appears to be the case due to inefficient substrate use[6]. In addition, methylotrophic methanogenesis produces more CH_4 than CO_2 at a ratio of 3:1 (reaction 5, here we use methanol as a representative substrate for methylotrophic methanogenesis) but typically contributes only a minor portion of CH_4 production (< 5%) in freshwater sediments[15,16]. Furthermore, the proportion of methylotrophic methanogens in freshwater sediments is typically small (<10%, see refs[17–20] as examples) which agrees with what we found here with the methylotrophic methanogens constituting ~9% of the methanogen sequences in our streambeds (see Supplementary Figure 3). Note, the hydrogen-dependent methylotrophs accounted for an even smaller proportion of the methylotrophs (12.4% of 9%) and, therefore, were not likely to influence the measured increase in CH_4:CO_2.

Reaction	Stoichiometry	Metabolism
1	$C_6H_{12}O_6 + 2H_2O \rightarrow 2CH_3COOH + 2CO_2 + 4H_2$	Fermentation
2	$4H_2 + CO_2 \rightarrow 2H_2O + CH_4$	Hydrogenotrophic
3	$2CH_3COOH \rightarrow 2CO_2 + 2CH_4$	Acetoclastic
4	$C_6H_{12}O_6 \rightarrow 3CH_4 + 3CO_2$ (1:1 ratio)	Sum of 1 to 3
5	$4CH_3OH \rightarrow 3CH_4 + 1CO_2 + 2H_2O$ (3:1 ratio)	Methylotrophic

Supplementary Table 3 | Hydrophysical characteristics for the streams in the five high-latitude regions. Data are presented as median, first (Q1) and third (Q3) quartiles.

**Region**	**Hydrophysical characteristics** Median (Q1-Q3)			
	**Width** (m)	**Depth** (m)	**Flow** (m s^{-1})	$k_{O2\_20°C}$ (cm h^{-1})
Alaska (Manley Hot Springs)	1.0 (0.71-1.5)	0.2 (0.13-0.46)	0.24 (0.13-0.43)	33.6 (15.4-50.0)
Greenland (Qeqertarsuaq/ Disko Island)	0.44 (0.29-0.68)	0.1 (0.05-0.14)	0.33 (0.06-0.69)	24.1 (20.4-31.5)
Iceland (Hengill Valley)	0.87 (0.63-1.5)	0.09 (0.07-0.11)	0.28 (0.17-0.45)	72.2 (50.6-112)
Kamchatka[††] (Verkhne-Paratunskiye thermal springs)	0.58 (0.45-0.83)	0.04 (0.03-0.054)	0.37 (0.16-0.41)	125 (33.9-169)
Svalbard (North-Western Spitsbergen National Park)	0.48 (0.3-0.61)	0.06 (0.04-0.08)	0.13 (0.06-0.16)	80.6 (38.4-84.0)

Supplementary Table 4 | Chemical characteristics for the streams in the five high-latitude regions. Data are presented as median, first (Q1) and third (Q3) quartiles.

**Region**	**Chemical characteristics of surface stream water** Median (Q1-Q3)					**Chemical characteristics of sediment porewater**††† Median (Q1-Q3)	
	**pH**	**TIN**† (µmol L^{-1})	**SRP**† (µmol L^{-1})	**Dissolved CH_4** (nmol L^{-1})	**Dissolved O_2** (µmol L^{-1})	**Dissolved CH_4** (nmol L^{-1})	**Dissolved O_2** (µmol L^{-1})
Alaska (Manley Hot Springs)	7.6 (7.4-7.8)	2.2 (2.1-2.5)	0.93 (0.48-2.0)	174 (87-279)	325 (261-347)	155 (105-2670)	238 (154-317)
Greenland (Qeqertarsuaq/ Disko Island)	8.4 (7.8-10)	1.1 (0.8-1.6)	0.83 (0.54-0.96)	31 (18-68)	368 (268-399)	540 (95-4630)	250 (172-349)
Iceland (Hengill Valley)	8.1 (7.6-8.2)	0.8 (0.6-0.9)	1.1 (0.82-1.3)	41 (29-65)	302 (272-333)	114 (93-148)	256 (187-313)
Kamchatka†† (Verkhne-Paratunskiye thermal springs)	7.5 (7.3-7.6)	5.7 (4.3-6.7)	4.2 (2.8-4.6)	35 (28-38)	330 (277-354)	144 (126-384)	265 (236-311)
Svalbard (North-Western Spitsbergen National Park)	7.9 (7.3-8.5)	4.4 (2.8-15.5)	1.3 (0.68-1.4)	43 (35-47)	330 (284-385)	NA	NA

†Total inorganic nitrogen (TIN) is the sum of NO_3^-, NO_2^-, NH_4^+ and SRP is soluble reactive phosphorus (see Supplementary Methods). See Supplementary Figure 2 for a comparison with streams across continental Europe.
††While Kamchatka is at a lower latitude than the other four regions, its snow-cover, stream temperature and remoteness sustain a similar climate.
†††Porewater collected from 2 to 10 cm depth. Porewater probes could not be deployed in Svalbard, therefore no porewater CH_4 concentrations could be determined in this region.

Supplementary Table 5 | Parameter estimates to characterize the temperature sensitivity in methane-related processes. Mean parameter estimates, 95% credible intervals for lower (2.5%) and upper (97.5%) bound, effective sample size (Eff), and the Gelman-Rubin statistic ($\hat{R}$), were obtained using a Bayesian hierarchical model. See Methods for parameter notation and model fitting approach. Overall region-level model fits are visually demonstrated in Extended Data Figure 5.

(5a) For CH_4 emission:

	**Parameter**	**Mean**	**2.5%**	**97.5%**	**Eff**	$\hat{R}$
Regression coefficients	α	-0.04	-0.54	0.45	2559	1.00
	β	0.58	0.25	0.93	3361	1.00
Region-level parameters	$\sigma(a_{0j})$	0.14	0.00	0.48	3955	1.00
	$\sigma(a_{1j})$	0.20	0.01	0.54	1985	1.00
	$Cor(a_{0j}, a_{1j})$	0.06	-0.79	0.84	4882	1.00
Stream-level parameters	$\sigma(b_{0jk})$	1.09	0.84	1.40	2876	1.00
	$\sigma(b_{1jk})$	0.14	0.00	0.45	1547	1.00
	$Cor(b_{0jk}, b_{1jk})$	0.25	-0.70	0.91	4435	1.00
Distributional parameter	σ	0.80	0.70	0.93	5048	1.00

(5b) For CH_4 production:

	**Parameter**	**Mean**	**2.5%**	**97.5%**	**Eff**	$\hat{R}$
Regression coefficients	α	-4.34	-5.02	-3.46	3050	1.00
	β	0.73	0.31	1.13	4353	1.00
Region-level parameters	$\sigma(a_{0j})$	0.53	0.03	1.17	1689	1.00
	$\sigma(a_{1j})$	0.14	0.00	0.48	4261	1.00
	$Cor(a_{0j}, a_{1j})$	-0.01	-0.81	0.80	7943	1.00
Stream-level parameters	$\sigma(b_{0jk})$	0.62	0.02	1.24	863	1.00
	$\sigma(b_{1jk})$	0.19	0.01	0.63	3190	1.00
	$Cor(b_{0jk}, b_{1jk})$	0.12	-0.74	0.85	6252	1.00
Distributional parameter	σ	1.34	1.06	1.70	1811	1.00

(5c) For CH_4 to CO_2 production ratio:

	**Parameter**	**Mean**	**2.5%**	**97.5%**	**Eff**	$\hat{R}$
Regression coefficients	α	-3.50	-5.19	-1.62	3748	1.00

	β	1.15	0.55	1.73	9701	1.00
Region-level parameters	$\sigma(a_{0j})$	2.02	1.39	2.87	5662	1.00
	$\sigma(a_{1j})$	0.16	0.00	0.57	6262	1.00
	$Cor(a_{0j}, a_{1j})$	-0.04	-0.82	0.80	10883	1.00
Stream-level parameters	$\sigma(b_{0jk})$	0.18	0.00	0.62	6288	1.00
	$\sigma(b_{1jk})$	0.16	0.00	0.56	7096	1.00
	$Cor(b_{0jk}, b_{1jk})$	0.00	-0.81	0.81	12570	1.00
Distributional parameter	σ	2.03	1.70	2.43	9513	1.00

(5d) For CH_4 oxidation:

	**Parameter**	**Mean**	**2.5%**	**97.5%**	**Eff**	$\hat{R}$
Regression coefficients	α	-0.09	-0.99	0.85	3850	1.00
	β	0.69	0.23	1.13	5321	1.00
Region-level parameters	$\sigma(a_{0j})$	0.79	0.09	1.48	1759	1.00
	$\sigma(a_{1j})$	0.17	0.01	0.58	5404	1.00
Stream-level parameters	$\sigma(b_{0jk})$	1.35	0.88	1.91	2013	1.00
Distributional parameter	σ	1.32	1.09	1.62	4339	1.00

(5e) For *mcrA* abundance:

	**Parameter**	**Mean**	**2.5%**	**97.5%**	**Eff**	$\hat{R}$
Regression coefficients	α	8.04	7.33	8.75	3470	1.00
	β	0.41	0.05	0.77	3704	1.00
Region-level parameters	$\sigma(a_{0j})$	0.64	0.20	1.18	2113	1.00
	$\sigma(a_{1j})$	0.15	0.00	0.50	3946	1.00
	$Cor(a_{0j}, a_{1j})$	-0.05	-0.81	0.77	8727	1.00
Stream-level parameters	$\sigma(b_{0jk})$	0.96	0.72	1.26	1829	1.00
	$\sigma(b_{1jk})$	0.17	0.01	0.57	1504	1.00
	$Cor(b_{0jk}, b_{1jk})$	0.00	-0.77	0.77	8087	1.00
Distributional parameter	Shape	2.53	1.81	3.36	6399	1.00
	z_i	0.27	0.21	0.34	14510	1.00

(5f) For *pmoA* abundance:

	**Parameter**	**Mean**	**2.5%**	**97.5%**	**Eff**	$\hat{R}$

Regression coefficients	α	7.97	7.50	8.46	2844	1.00
	β	0.51	0.17	0.84	2958	1.00
Region-level parameters	$\sigma(a_{0j})$	0.31	0.01	0.79	1186	1.00
	$\sigma(a_{1j})$	0.13	0.00	0.46	3115	1.00
Stream-level parameters	$\sigma(b_{0jk})$	0.92	0.70	1.18	2427	1.00
Distributional parameter	Shape	1.56	1.18	2.00	4969	1.00
	z_i	0.11	0.06	0.16	9895	1.00

Supplementary Table 6 | Subset of sample DNA concentrations quantified in extracts used as templates for downstream molecular work as evidence of the low biomass within sediment, ordered from highest to lowest.

Sample ID	DNA concentration (ng/µL)	Sample ID	DNA concentration (ng/µL)	Sample ID	DNA concentration (ng/µL)	Sample ID	DNA concentration (ng/µL)	Sample ID	DNA concentration (ng/µL)
B8	15.6	51	3.4	11	1.1	HD	0.4	34	<0.1
98	12.1	92	3.4	5	1.1	37	0.4	89	<0.1
87	11.3	32	3.4	30	1.0	59	0.3	101	<0.1
5.3	10.8	61	3.3	73	1.0	6	0.3	46	<0.1
B2	9.2	63	3.1	72	1.0	70	0.3	90	<0.1
108	7.3	50	2.9	17	0.9	A5	0.3	48	<0.1
54	7.5	93	2.9	100	0.8	A1	0.2	79	<0.1
B9	7.4	74	2.8	C2	0.8	58	0.2	57	<0.1
97	7.0	52	2.8	49	0.7	44	0.3	13	<0.1
B6	6.6	HB	2.7	33	0.7	A4	0.2	99	<0.1
12	6.4	104	2.4	22	0.7	25	0.2	7	<0.1
21	6.2	81	2.2	A7	0.8	110	0.2	36	<0.1
5.1	6.0	18	2.3	29	0.7	B7	0.2	35	<0.1
43	6.0	41	2.2	31	0.7	C7	0.2		
28	5.3	8	2.1	47	0.6	27	0.2		
85	5.0	B10	2.1	91	0.6	66	0.1		
83	4.7	19	2.0	38	0.6	HA	0.1		
20	4.9	53	1.9	14	0.3	88	0.1		
94	4.5	95	1.9	105	0.5	102	0.1		

Supplementary Table 7 | Parameter estimates to evaluate changes in community diversity and community compositional changes along the natural warming gradient. Mean parameter estimates, 95% credible intervals for lower (2.5%) and upper (97.5%) bound, effective sample size (Eff.), and the Gelman-Rubin statistic ($\hat{R}$), were obtained using a Bayesian hierarchical model. See Methods for parameter notation and model fitting approach.

Prior to model fitting, the NGS reads of *mcrA* sequences were rarefied to 1,000 reads per sample and the proportion of methanogen families (i.e., *Methanobacteriaceae*, *Methanocellaceae*, *Methanoregulaceae*, *Methanotrochaceae* and Methylotrophs, see Extended Data Figure 6) in each sample calculated as reads of family per 1,000 reads per sample. For *pmoA* sequences, the NGS reads were rarefied to 1,006 reads per sample (the minimum sample size of the *pmoA* library) and the proportion of methanotroph types (i.e., type I and type II, see Extended Data Figure 7) in each sample calculated as reads of type per 1,006 reads per sample. The methanogen or methanotroph communities were first assessed using Shannon index (**Tables 7a** and **7b**), followed by an analysis of the relative abundance of methanogen families and methanotroph types (**Table 7cs** and **7d**).

(7a) For Shannon index in *mcrA* community:

	Parameter	Mean	2.5%	97.5%	Eff.	$\hat{R}$
Regression coefficients	α	1.07	0.88	1.25	2839	1.00
	β	0.01	-0.18	0.18	2659	1.00
Region-level parameters	$\sigma(a_{0j})$	0.13	0.00	0.36	1924	1.00
	$\sigma(a_{1j})$	0.10	0.00	0.32	2496	1.00
	$Cor(a_{0j}, a_{1j})$	-0.10	-0.84	0.75	6086	1.00
Stream-level parameters	$\sigma(b_{0jk})$	0.27	0.16	0.38	2724	1.00
	$\sigma(b_{1jk})$	0.06	0.00	0.20	2003	1.00
	$Cor(b_{0jk}, b_{1jk})$	0.03	-0.78	0.82	5953	1.00
Distributional parameter	σ	0.32	0.28	0.37	5197	1.00

(7b) For Shannon index in *pmoA* community:

	Parameter	Mean	2.5%	97.5%	Eff.	$\hat{R}$
Regression coefficients	α	0.31	0.15	0.48	4061	1.00
	β	-0.04	-0.14	0.06	4410	1.00

	$\sigma(a_{0j})$	0.16	0.12	0.21	3745	1.00
Region-level parameters	$\sigma(a_{1j})$	0.04	0.00	0.12	1432	1.00
	$Cor(a_{0j}, a_{1j})$	0.17	-0.72	0.86	6255	1.00
	$\sigma(b_{0jk})$	0.16	0.06	0.36	3895	1.00
Stream-level parameters	$\sigma(b_{1jk})$	0.07	0.00	0.21	2577	1.00
	$Cor(b_{0jk}, b_{1jk})$	-0.02	-0.79	0.76	9697	1.00
Distributional parameter	σ	0.16	0.15	0.18	9191	1.00

(7c) For compositional changes in *mcrA* community:

	**Parameter**	**Mean**	**2.5%**	**97.5%**	**Eff.**	$\hat{R}$
Regression coefficients	α	0.31	0.25	0.37	3490	1.00
	β_1	0.03	-0.02	0.07	3641	1.00
	$\gamma_{g=Methanobacteriaceae}$	-0.04	-0.11	0.03	6097	1.00
	$\gamma_{g=Methanoregulaceae}$	0.31	0.25	0.38	6081	1.00
	$\gamma_{g=Methanotrichaceae}$	0.17	0.10	0.23	5796	1.00
	$\gamma_{g=\text{Methylotroph}}$	-0.03	-0.10	0.23	5796	1.00
	$\delta_{g=Methanobacteriaceae}$	0.03	-0.04	0.10	4706	1.00
	$\delta_{g=Methanoregulaceae}$	-0.13	-0.20	-0.06	4670	1.00
	$\delta_{g=Methanotrichaceae}$	0.01	-0.06	0.08	5057	1.00
	$\delta_{g=\text{Methylotroph}}$	-0.04	-0.11	0.03	4799	1.00
Region-level parameters	$\sigma(a_{0j})$	0.03	0.00	0.09	2436	1.00
Stream-level parameters	$\sigma(b_{0jk})$	0.01	0.00	0.03	6022	1.00
Distributional parameter	σ	0.27	0.26	0.29	14589	1.00

Note that the terms δ_g are the category-specific temperature interaction effects relative to the baseline (β_1, corresponding to *Methanocellaceae*; see Eq.21 in the Methods). The slopes (β) and their credible intervals shown in Fig.3B and 3C correspond to the overall estimated slopes for each taxonomic category, derived from the posterior draws. The posterior probabilities for the slopes being positive or negative were assessed using one-sided hypothesis testing and are measures of Bayesian evidence supporting directional effects.

(7d) For compositional changes in *pmoA* community:

	Parameter	Mean	2.5%	97.5%	Eff.	$\widehat{R}$
Regression coefficients	α	0.51	0.44	0.57	6271	1.00
	β_1	-0.06	-0.12	0.00	6161	1.00
	$\gamma_{g=\text{type II}}$	0.56	0.48	0.65	7881	1.00
	$\delta_{g=\text{ type II}}$	0.12	0.04	0.21	6066	1.00
Region-level parameters	$\sigma(a_{0\text{j}})$	0.03	0.00	0.09	4016	1.00
Stream-level parameters	$\sigma(b_{0\text{jk}})$	0.02	0.00	0.05	5830	1.00
Distributional parameter	σ	0.42	0.40	0.45	10812	1.00

Note that the terms δ_g is the temperature interactions of type II methanotroph relative to baseline (β_1, corresponding to type I methanotroph, see Eq.21 in the Methods). The slopes (β) and their credible intervals shown in Fig.3E and 3F correspond to the overall estimated slopes for each methanotroph type, derived from posterior draws. The posterior probabilities for the slopes being positive or negative were assessed using one-sided hypothesis testing and are measures of Bayesian evidence supporting directional effects.

Supplementary Table 8 | Parameter estimates to evaluate process-level CH_4 oxidation efficiency and system-level CH_4 filter efficiency. Mean parameter estimates, 95% credible intervals for lower (2.5%) and upper (97.5%) bound, effective sample size (Eff.), and the Gelman-Rubin statistic ($\hat{R}$), were obtained using a Bayesian hierarchical model. See Methods for parameter notation and model fitting approach.

(8a) For process-level CH_4 oxidation efficiency:

	**Parameter**	**Mean**	**2.5%**	**97.5%**	**Eff.**	$\hat{R}$
Regression coefficients	α	-0.10	-1.33	1.11	6570	1.00
	β	0.25	-0.79	1.30	8210	1.00
Region-level parameters	$\sigma(a_{0j})$	0.99	0.25	1.72	2642	1.00
	$\sigma(a_{1j})$	0.23	0.01	0.85	7618	1.00
	$Cor(a_{0j}, a_{1j})$	0.12	-0.75	0.86	15871	1.00
Stream-level parameters	$\sigma(b_{0jk})$	1.12	0.24	1.79	1717	1.00
	$\sigma(b_{1jk})$	0.22	0.01	0.85	7752	1.00
	$Cor(b_{0jk}, b_{1jk})$	0.07	-0.77	0.83	15615	1.00
Distributional parameter	σ	1.52	1.21	1.98	2824	1.00

Note that the term β is the effect of warm streams relative to cold streams α (see Eq. 23 in the Methods). The process-level CH_4 oxidation efficiency in the cold and warm streams and their credible intervals reported in Fig.4C in the main text are estimated from posterior draws.

(8b) For system-level CH_4 filter efficiency:

	**Parameter**	**Mean**	**2.5%**	**97.5%**	**Eff.**	$\hat{R}$
Regression coefficients	α	1.09	0.47	1.71	3065	1.00
	β	0.02	-0.84	0.87	3479	1.00
Region-level parameters	$\sigma(a_{0j})$	0.21	0.01	0.67	2666	1.00
	$\sigma(a_{1j})$	0.22	0.01	0.81	4093	1.00
	$Cor(a_{0j}, a_{1j})$	0.00	-0.81	0.81	10471	1.00
Stream-level parameters	$\sigma(b_{0jk})$	1.01	0.67	1.41	2963	1.00
	$\sigma(b_{1jk})$	0.19	0.01	0.69	3556	1.00
	$Cor(b_{0jk}, b_{1jk})$	0.03	-0.77	0.82	11022	1.00
Distributional parameter	φ	11.50	7.11	16.86	4508	1.00

Note that, as the system-level CH_4 filter efficiency is a continuous proportion bounded between 0 and 1, we fitted a beta regression model with logit link (see Eq.24 in the Methods). Therefore, the numbers reported here are on the logit-transformed scale. The efficiency estimates and credible intervals presented in Fig. 4D were derived by applying the inverse logit transformation to posterior draws. For example, α - the intercept representing the process-level CH_4 oxidation efficiency for cold streams – is 1.09 on the logit scale. Applying the inverse logit transformation: $\frac{e^{1.09}}{1+e^{1.09}}$=0.75, i.e., corresponds to 75% efficiency. Similarly, β is the effect of warm streams relative to cold streams on the logit scale; combined with the intercept, this gives: $\frac{e^{(1.09+0.02)}}{1+e^{(1.09+0.02)}}$=0.75, i.e., 75% efficiency for warm streams.

Supplementary Table 9 | Primer sets for qPCR and NGS.

A, Primer sets used in both qPCR and NGS library preparation to target methanogenic Archaea (*mcrA*) and methanotrophic Bacteria (*pmoA*) with both primer sets containing the same locus specific regions. The primer sets used for NGS include Illumina overhang adapters for binding to the Illumina flow cell[23].

Target gene	Primer name	Primer sequence (5'-3')
mcrA (Methyl coenzyme M reductase)	mlas-mod[†24,25]	5'-GGYGGTGTMGGDTTCACMCARTA-3'
	mcrA-rev[25]	5'-CGTTCATBGCGTAGTTVGGRTAGT-3'
pmoA (Particulate methane monooxygenase)	A189[26]	5'-GGNGACTGGGACTTCTGG-3'
	A650[27]	5'- ACGTCCTTACCGAAGGT-3'

[†] *mlas-mod* is only 1 bp different from original *mlas* forward primer[25]

Use of qPCR to estimate methanogen and methanotroph abundance. Steinberg and Regan (2009)[28] examined the complete genomes of 19 methanogens submitted to GenBank and found only one copy of *mcrA* in all 19 and we could find no evidence of multiple copies of *mcrA* in the 356 papers citing them since. Further, while members of the *Methanococcales* and *Methanobacteriales* do also contain a copy of the *mrtA* variant of *mcrA* we could find no evidence from all of our sequences to indicate any amplification of *mrtA*, nor in the literature that used the same primer pair as we did – see Emilson *et al*. 2018 (ref.[29]); Zhang *et al*. 2020 (ref.[30]) as examples. In contrast, it is recognised that the genomes of some methanotrophs do contain multiple copies of *pmoA* (**B,** below). We searched in KEGG, the genome database and individual published species descriptions for *pmoA* copy number (below and see literature list provided as a separate supporting file) and found copy numbers in type I methanotrophs to be like those for type II methanotrophs, with an overall average of 1.5 which is close to the 1.62 reported previously[31].

Genus	Type	Species	Strains	Range of copy number	Average copy number
Methylomonas	Type I	27	31	1-3	1.6
Methylobacter	Type I	7	10	1-2	1.8
Methylococcus	Type I	3	3	2	2
Methylocaldum	Type I	2	2	2	2
Methylomicrobium	Type I	6	7	1-2	1.3

Methylosarcina	Type I	1	2	1	1
**Average across type I genus**					**1.6**
Methylosinus	Type II	1	1	1	1
Methylocystis	Type II	9	9	1-3	2.2
Methylocapsa	Type II	3	1	1	1
**Average across type II genus**					**1.4**
**Average across types**					**1.5**

As *pmoA* copy number was comparable between the type I and type II methanotrophs we used the overall average of 1.5 across the two types to correct the increase in methanotroph abundance in response to warming presented in Fig. 4B in the main text and below. Note that while the correction to *pmoA* copy number (panel B, below) reduced the average estimate of methanotroph abundance (i.e., the intercept at T_c) the response to warming i.e., their temperature sensitivity (slope) remained the same at 0.51 eV.

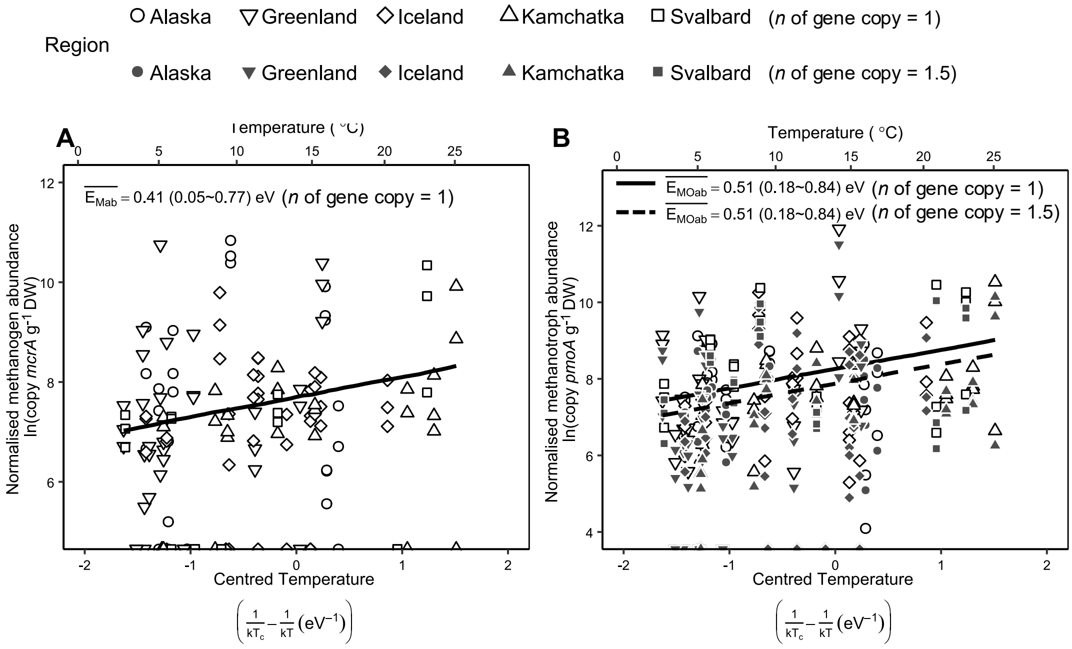

References

1. Kirkwood, D. Nutrients: Practical notes on their determination in sea water. *ICES Tech. Mar. Environ. Sci.* **17**, 25 (1996).
2. Si, Y. *et al.* Direct biological fixation provides a freshwater sink for N_2O. *Nat. Commun.* **14**, 6775 (2023).
3. Chang, K.-Y., Riley, W. J., Crill, P. M., Grant, R. F. & Saleska, S. R. Hysteretic temperature sensitivity of wetland CH_4 fluxes explained by substrate availability and microbial activity. *Biogeosciences* **17**, 5849–5860 (2020).
4. Yvon-Durocher, G. *et al.* Methane fluxes show consistent temperature dependence across microbial to ecosystem scales. *Nature* **507**, 488–91 (2014).
5. Zhu, Y. *et al.* Separating natural from human enhanced methane emissions in headwater streams. *Nat. Commun.* **13**, 3810 (2022).
6. Zhu, Y., Purdy, K. J., Martínez Rodríguez, A. & Trimmer, M. A rationale for higher ratios of CH_4 to CO_2 production in warmer anoxic freshwater sediments and soils. *Limnol. Oceanogr. Lett.* **8**, 398–405 (2023).
7. Rovelli, L. *et al.* Contrasting biophysical controls on carbon dioxide and methane outgassing from streams. *J. Geophys. Res. Biogeosciences* **1**, 2–31 (2021).
8. Langenegger, T., Vachon, D., Donis, D. & McGinnis, D. F. What the bubble knows: Lake methane dynamics revealed by sediment gas bubble composition. *Limnol. Oceanogr.* **64**, 1526–1544 (2019).
9. Zhu, Y. *et al.* Disproportionate increase in freshwater methane emissions induced by experimental warming. *Nat. Clim. Change* **10**, 685–690 (2020).
10. Patel, L., Singh, R., Gowd, S. C. & Thottathil, S. D. Environmental determinants of aerobic methane oxidation in a tropical river network. *Water Res.* **265**, 122257 (2024).

11. Matoušů, A., Osudar, R., Šimek, K. & Bussmann, I. Methane distribution and methane oxidation in the water column of the Elbe estuary, Germany. *Aquat. Sci.* **79**, 443–458 (2017).

12. Rosentreter, J. A. *et al.* Half of global methane emissions come from highly variable aquatic ecosystem sources. *Nat. Geosci.* **14**, 225–230 (2021).

13. Hausfather, Z. & Peters, G. P. Emissions – the 'business as usual' story is misleading. *Nature* **577**, 618–620 (2020).

14. Conrad, R. Contribution of hydrogen to methane production and control of hydrogen concentrations in methanogenic soils and sediments. *FEMS Microbiol. Ecol.* **28**, 193–202 (1999).

15. Conrad, R. & Claus, P. Contribution of methanol to the production of methane and its ^{13}C-isotopic signature in anoxic rice field soil. *Biogeochemistry* **73**, 381–393 (2005).

16. Lovley, D. R. & Klug, M. J. Methanogenesis from methanol and methylamines and acetogenesis from hydrogen and carbon dioxide in the sediments of a eutrophic lake. *Appl. Environ. Microbiol.* **45**, 1310–1315 (1983).

17. Rasmussen, A. N., Tolar, B. B., Bargar, J. R., Boye, K. & Francis, C. A. Diverse and unconventional methanogens, methanotrophs, and methylotrophs in metagenome-assembled genomes from subsurface sediments of the Slate River floodplain, Crested Butte, CO, USA. *mSystems* **9**, e00314-24 (2024).

18. Narrowe, A. B. *et al.* Uncovering the Diversity and Activity of Methylotrophic Methanogens in Freshwater Wetland Soils. *mSystems* **4**, 10.1128/msystems.00320-19 (2019).

19. Söllinger, A. *et al.* Phylogenetic and genomic analysis of *Methanomassiliicoccales* in wetlands and animal intestinal tracts reveals clade-specific habitat preferences. *FEMS Microbiol. Ecol.* **92**, fiv149 (2016).

20. Ellenbogen, J. B. *et al.* Methylotrophy in the Mire: direct and indirect routes for methane production in thawing permafrost. *mSystems* **9**, e00698-23 (2024).

21. Brooks, M. E. *et al.* glmmTMB balances speed and flexibility among packages for zero-inflated generalized linear mixed modeling. *R J.* **9**, 378–400 (2017).

22. Lenth, R. emmeans: Estimated Marginal Means, aka Least-Squares Means. (2019).

23. Illumina. 16S Metagenomic Sequencing Library. *Illumina.com* 1–28 (2013).

24. Angel, R., Claus, P. & Conrad, R. Methanogenic archaea are globally ubiquitous in aerated soils and become active under wet anoxic conditions. *Isme J.* **6**, 847 (2011).

25. Steinberg, L. M. & Regan, J. M. Phylogenetic comparison of the methanogenic communities from an acidic, oligotrophic fen and an anaerobic digester treating municipal wastewater sludge. *Appl. Environ. Microbiol.* **74**, 6663–6671 (2008).

26. Holmes, A. J., Costello, A., Lidstrom, M. E. & Murrell, J. C. Evidence that participate methane monooxygenase and ammonia monooxygenase may be evolutionarily related. *FEMS Microbiol. Lett.* **132**, 203–208 (1995).

27. Bourne, D. G., McDonald, I. R. & Murrell, J. C. Comparison of *pmoA* PCR Primer Sets as Tools for Investigating Methanotroph Diversity in Three Danish Soils. *Appl. Environ. Microbiol.* **67**, 3802–3809 (2001).

28. Steinberg, L. M. & Regan, J. M. *mcrA*-targeted real-time quantitative PCR method to examine methanogen communities. *Appl. Environ. Microbiol.* **75**, 4435–4442 (2009).

29. Emilson, E. J. S. *et al.* Climate-driven shifts in sediment chemistry enhance methane production in northern lakes. *Nat. Commun.* **9**, 1801 (2018).

30. Zhang, C.-J., Pan, J., Liu, Y., Duan, C.-H. & Li, M. Genomic and transcriptomic insights into methanogenesis potential of novel methanogens from mangrove sediments. *Microbiome* **8**, 94 (2020).

31. Nijman, T. P. A. *et al.* Warming and eutrophication interactively drive changes in the methane-oxidizing community of shallow lakes. *ISME Commun.* **1**, 32 (2021).
