# Supplementary material for: A fixed methane filter maximizes freshwater emissions under warming
Source: Nat Clim Chang. 2026 Jun 5;16(6):704–11. doi: 10.1038/s41558-026-02649-2 (PMC13249575; doi:10.1038/s41558-026-02649-2)
Supplement: Supplementary file 2 — Reporting Summary [file 41558_2026_2649_MOESM2_ESM.pdf]

nature portfolio

Corresponding author(s): Mark Trimmer

Last updated by author(s): Apr 7, 2026

Reporting Summary

Nature Portfolio wishes to improve the reproducibility of the work that we publish. This form provides structure for consistency and transparency in reporting. For further information on Nature Portfolio policies, see our Editorial Policies and the Editorial Policy Checklist.

Statistics

For all statistical analyses, confirm that the following items are present in the figure legend, table legend, main text, or Methods section.

n/a	Confirmed	
☐	☒	The exact sample size (n) for each experimental group/condition, given as a discrete number and unit of measurement
☐	☒	A statement on whether measurements were taken from distinct samples or whether the same sample was measured repeatedly
☐	☒	The statistical test(s) used AND whether they are one- or two-sided<br>*Only common tests should be described solely by name; describe more complex techniques in the Methods section.*
☒	☐	A description of all covariates tested
☒	☐	A description of any assumptions or corrections, such as tests of normality and adjustment for multiple comparisons
☐	☒	A full description of the statistical parameters including central tendency (e.g. means) or other basic estimates (e.g. regression coefficient) AND variation (e.g. standard deviation) or associated estimates of uncertainty (e.g. confidence intervals)
☒	☐	For null hypothesis testing, the test statistic (e.g. F, t, r) with confidence intervals, effect sizes, degrees of freedom and P value noted<br>*Give P values as exact values whenever suitable.*
☐	☒	For Bayesian analysis, information on the choice of priors and Markov chain Monte Carlo settings
☒	☐	For hierarchical and complex designs, identification of the appropriate level for tests and full reporting of outcomes
☒	☐	Estimates of effect sizes (e.g. Cohen's d, Pearson's r), indicating how they were calculated

Our web collection on statistics for biologists contains articles on many of the points above.

Software and code

Policy information about availability of computer code

Data collection	Data for CH4 and CO2 concentration were collected using "GC ChemStation Software rev A.10.02". Quantification of methanogenic (mcrA) and methanotrophic (pmoA) copy numbers were collected using a CFX384TM Real-Time PCR Detection System (BIO-RAD, CA, US). Amplicon sequencing was performed on an Illumina MiSeq (600 cycles; reagent kit v3).
Data analysis	All statistical analyses were performed in R (version 4.2.3) and all graphical presentations were created using the “ggplot2” package (version 3.4.4). Bayesian models were fitted using the "brms" package (version 2.23.0). Model validation and comparison were performed using the "loo" package (version 2.8.0). Posterior analysis and visualization were conducted with the "posterior" (version 1.6.1), "tidybayes" (version 3.8.7), and "bayesplot" (version 1.14.0) packages.

For manuscripts utilizing custom algorithms or software that are central to the research but not yet described in published literature, software must be made available to editors and reviewers. We strongly encourage code deposition in a community repository (e.g. GitHub). See the Nature Portfolio guidelines for submitting code & software for further information.

Data

Policy information about availability of data

All manuscripts must include a data availability statement. This statement should provide the following information, where applicable:

- Accession codes, unique identifiers, or web links for publicly available datasets
- A description of any restrictions on data availability
- For clinical datasets or third party data, please ensure that the statement adheres to our policy

Source data are provided with this manuscript. Raw sequencing data for both methanogens and methanotrophs are available via NCBI SRA online repository under the BioProject number numbers PRJNA1119395 for methanogens and PRJNA1119373 for methanotrophs.

Research involving human participants, their data, or biological material

Policy information about studies with human participants or human data. See also policy information about sex, gender (identity/presentation), and sexual orientation and race, ethnicity and racism.

Reporting on sex and gender	No human participants were involved in this study.
Reporting on race, ethnicity, or other socially relevant groupings	N/A
Population characteristics	N/A
Recruitment	N/A
Ethics oversight	N/A

Note that full information on the approval of the study protocol must also be provided in the manuscript.

Field-specific reporting

Please select the one below that is the best fit for your research. If you are not sure, read the appropriate sections before making your selection.

☐ Life sciences ☐ Behavioural & social sciences ☒ Ecological, evolutionary & environmental sciences

For a reference copy of the document with all sections, see nature.com/documents/nr-reporting-summary-flat.pdf

Ecological, evolutionary & environmental sciences study design

All studies must disclose on these points even when the disclosure is negative.

Study description	In this study, we used 52 ambient and naturally warmed (1 to 36 °C) streams across five high-latitude regions (in Iceland, Alaska, Greenland, Svalbard and Kamchatka) spanning the Northern Hemisphere as a unique test of the natural effects of warming on the CH4 filter. We showed that streambed methanogenesis became more efficient with natural warming with fundamental changes in methanogen community composition. In contrast, methanotroph communities became dominated by less efficient methanotrophs and overall failed to constrain CH4 emissions temperature increased. Our study provided intercontinental-scale evidence that the capacity of the CH4 filter appears to be capped under warming and if the "capping" is common to natural sources of CH4 worldwide, a positive feedback loop accelerating future warming on Earth appears inevitable.
Research sample	We visited geothermal catchments in the 5 regions of Iceland, Alaska, Greenland, Svalbard and Kamchatka where we typically sampled 7 to 13 streams per region to give us 52 streams in total. Here, indirect warming through the bedrock generated a natural temperature gradient in the streams from 1 to 36 °C, a typical temperature range of natural CH4 sources (wetlands, lakes, streams, for example) on Earth. These naturally warmed streams are off any anthropogenic influence, are circumneutral and share comparable hydrophysical and chemical characteristics.<br>Samples for hydrological and chemical properties of the streams were determined within a 50 m transect. Porewater samples were collected using probes inserted at 2, 4, 6, 8 and 10 cm depth where the streambeds were penetrable. Surface water samples for dissolved gases were collected at three equally spaced locations along each stream transect. Sediment samples for CH4 production and CH4 oxidation potentials were collected from the surface streambed sediments. To link the microbial community and abundance to related potentials, sediments for molecular microbial analyses were also collected.
Sampling strategy	No prior statistical analysis was performed to determine sample size. However, across the five high-latitude regions mentioned above we typically sampled 7 to 13 streams per region, which is robust for regression, to give a total of 52 streams. This sampling strategy provided a natural thermal gradient in the streams from 1 to 36 °C, in total, covering a temperature range of typical natural sources of CH4 on Earth. Further, technical triplicates were collected in each stream providing a dataset suitable for fitting hierarchical Boltzmann-Arrhenius models in a fully Bayesian framework where the variations across streams or regions were accounted for. This

approach allowed us to estimate the overall temperature sensitivity of CH4 emission, CH4 production, CH4 oxidation etc, and to evaluate these relationship across the 5 regions.

Data collection	Data for hydrophysical and chemical characteristics were collected using sensors connected to a multiprobe and stream temperature data were collected using MiniDots. The data above were collected in situ by SFH with assistance from other members of the field team.<br>Data for gas concentrations and potentials of CH4 production and oxidation were collected using a gas chromatograph Agilent Technology UK Ltd., South Queensferry, UK) and recorded by the chromatography data system "GC ChemStation Software rev A.10.02". Data for total inorganic nitrogen and soluble reactive phosphorous were collected using an automated wet chemistry analyzer (San++, SKALAR Analytical B.V.) and recorded by software "FlowAccess". The data described above were collected by SFH.<br>Data for methanogen and methanotroph abundance were collected using a CFX384TM Real-Time PCR Detection System (BIO-RAD, CA, US) by KR. Data for methanogen and methanotroph community composition were collected on an Illumina MiSeq (600 cycles; reagent kit v3) via the NERC Biomolecular Analysis Facility at the Centre for Genome Research (Liverpool, UK) after sample preparation by KR.
Timing and spatial scale	Field sampling was performed over two summers in 2016 and 2017: Hengill Valley in Iceland; Manley Hot Springs in Alaska; Disko Island in Greenland; the North-Western Spitsbergen National Park in Svalbard; and the Verkhne-Paratunskiye thermal springs in Kamchatka, Russia. The coordinates of each sampling region are provided in Supplementary Table 3 and 4.<br>At each sampling site, data for hydrophysical and chemical characteristics were collected during the daytime while the MiniDot loggers were placed within each stream for a minimum of 24 hours to continuously record temperature. Sediment samples for potential of CH4 production and oxidation were collected and transferred to gas-tight vials during the daytime, flushed with OFN for production or left with air for oxidation and then incubated for the next 48 hours before being fixed with formaldehyde.
Data exclusions	Typically 7 to 13 streams were visited in each sampling region to give 52 streams in total. Due to difficulties in sampling streambeds armored with cobbles in Svalbard and some additional samples being lost in the field or during transportation, the datasets for CH4 production and oxidation potentials (in Fig. 2A , 2B and 4A) and the capacity of the CH4 filter (in Fig. 4C and 4D) were incomplete. Further, CH4 emissions for 2 streams fell outside the 95% percentiles for the dataset and were recognised as outliers and were therefore excluded from the emissions analysis (Fig. 1D).
Reproducibility	Across the five high-latitude regions spanning the Northern Hemisphere, a total of 52 streams were visited with typically 7 to 13 streams being sampled in each region. The combination of study sites provided a natural warming experiment using streams (well-known sources for CH4 worldwide) across a typical temperature range for natural aquatic CH4 sources on Earth (1 to 36 °C). Further, technical triplicates were collected at each sampled stream to form a complete dataset suitable for analysing the temperature sensitivity of each component in the CH4 cycle (methanogenic production, methanotrophy, the CH4 filter and CH4 emissions) using hierarchical Bayesian models. All the disclosure mentioned above guaranteed a reproducible and representable result.
Randomization	The sampling transect for each stream sampled was randomly selected. The incubation vials were randomly ordered for deoxygenation or for the addition of CH4. Samples for gas concentration, CH4 production and CH4 oxidation were analysed in random order.
Blinding	The data collection for the in-situ process rate measurement activities was led by SFH, as part of their postdoc, in the field with assistance from other members of the field team or in the laboratory largely by SFH solely. As such blinding was not applicable. Similarily, KR led the field sampling of sediment samples for molecular characterisation and processed sediment samples for DNA extraction, downstream qPCR assays and amplicon library preparation as part of their postdoc. Hence, again, blinding was not applicable.

Did the study involve field work? ☒ Yes ☐ No

Field work, collection and transport

Field conditions	As described above, field work was performed over the course of two summers in 2016 and 2017 and the annual mean air temperature of these five sampling regions ranged from ~ -8 to 4 °C. The temperature of the study streams ranged from 1 to 36 °C overall and the hydrophysical and chemical characteristics of the streams are provided in Supplementary Table 3 and 4.
Location	The coordinates of the sampling regions and hydrophysical characteristics of the study streams are provided in Supplementary Table 3.
Access & import/export	Local scientists were involved in field work. No special permits were required for access to the sampling locations.
Disturbance	We minimised trampling by using the same path to sites. No other disturbance was caused.

Reporting for specific materials, systems and methods

We require information from authors about some types of materials, experimental systems and methods used in many studies. Here, indicate whether each material, system or method listed is relevant to your study. If you are not sure if a list item applies to your research, read the appropriate section before selecting a response.

Materials & experimental systems

n/a	Involved in the study
☒	☐ Antibodies
☒	☐ Eukaryotic cell lines
☒	☐ Palaeontology and archaeology
☒	☐ Animals and other organisms
☒	☐ Clinical data
☒	☐ Dual use research of concern
☒	☐ Plants

Methods

n/a	Involved in the study
☒	☐ ChIP-seq
☒	☐ Flow cytometry
☒	☐ MRI-based neuroimaging

Plants

Seed stocks	N/A
Novel plant genotypes	N/A
Authentication	N/A
