# Supplementary material for: A fixed methane filter maximizes freshwater emissions under warming
Source: Nat Clim Chang. 2026 Jun 5;16(6):704–11. doi: 10.1038/s41558-026-02649-2 (PMC13249575; doi:10.1038/s41558-026-02649-2)
Supplement: Supplementary file 3 — Peer Review File [file 41558_2026_2649_MOESM3_ESM.pdf]

nature portfolio

Peer Review File

A fixed methane filter maximises freshwater emissions under warming

Corresponding Author: Professor Mark Trimmer

This manuscript has been previously reviewed at another journal. This document only contains information relating to versions considered at Nature Climate Change.

Version 0:

Reviewer comments:

Reviewer #1

(Comments for the Author)
The authors have done an excellent job revising the manuscript. I appreciate the hard work that went into this revision. My previous questions were addressed satisfactorily.

I have few remaining comments. One general question that I did not think of when I first reviewed the paper is, given these are geothermal systems, what is the potential influence of sulfate on methane cycling? High sulfate availability suppresses methanogenesis in marine environments and inland hard water ecosystems. Can the authors rule out such a confounding effect? I presume they do not have sulfate concentration data (if they do, then that is great). Do they have specific conductivity measurements from the streams which could be added to the PCA? If not, can they demonstrate from the literature that sulfate scales with pH, for instance, and pH is orthogonal to methane in the PCA? Some exploration of this issue would be useful.

My specific comments are all minor:

Line 107 - grammar
Line 148 - odd phrasing - not an example - this is the beginning of your mechanistic explanation
Line 181 - hydrogenotroph, singular
Line 181 - this is not an example, so reword
Line 251 - new subheading here?
Line 323-4 - grammar
Line 688 - slope, not slop

Line 385 - scale bar is cut off at top of figure

Reviewer #2

(Comments for the Author)
The authors have done a great and thorough job in the answers to my comments and in this revision. This work is novel, interesting and highly important. Thank you.
I have only two very minor comments:

Line 78. “temperature was a key environmental factor”. In what was temp the key environmental factor?

Line 641. Should be probably MUSCLE aligner? Not MUCLE

Open Access This Peer Review File is licensed under a Creative Commons Attribution 4.0 International License, which permits use, sharing, adaptation, distribution and reproduction in any medium or format, as long as you give appropriate credit to the original author(s) and the source, provide a link to the Creative Commons license, and indicate if changes were made.
In cases where reviewers are anonymous, credit should be given to 'Anonymous Referee' and the source.
The images or other third party material in this Peer Review File are included in the article’s Creative Commons license, unless indicated otherwise in a credit line to the material. If material is not included in the article’s Creative Commons license and your intended use is not permitted by statutory regulation or exceeds the permitted use, you will need to obtain permission directly from the copyright holder.
To view a copy of this license, visit https://creativecommons.org/licenses/by/4.0/

NCLIM-26010169-T Final reviewers’ comments

Reviewer #1 (Comments for the Author):

The authors have done an excellent job revising the manuscript. I appreciate the hard work that went into this revision. My previous questions were addressed satisfactorily.

I have few remaining comments. One general question that I did not think of when I first reviewed the paper is, given these are geothermal systems, what is the potential influence of sulfate on methane cycling? High sulfate availability suppresses methanogenesis in marine environments and inland hard water ecosystems. Can the authors rule out such a confounding effect? I presume they do not have sulfate concentration data (if they do, then that is great). Do they have specific conductivity measurements from the streams which could be added to the PCA? If not, can they demonstrate from the literature that sulfate scales with pH, for instance, and pH is orthogonal to methane in the PCA? Some exploration of this issue would be useful.

With hindsight we should have more fully explained the nature of our geothermal streams in the main text, rather than just citing papers from two of our co-authors who have worked in this model, geothermal system previously (Jackson et al. 2024; O'gorman et al. 2014). Our geothermal streams are warmed indirectly through the bedrock, rather than directly like the waters in Yellow Stone Park that have been used extensively to study extremophiles. This unique characteristic of our geothermal streams enables us to isolate the effect of temperature without the confounding effects of extreme water chemistries as in Yellow Stone Park and elsewhere. As detailed in (Jackson et al. 2024) there is no correlation between conductivity and temperature and only a weak negative correlation (-0.42) between pH and conductivity, which is principally driven by Svalbard as an outlier. We have added this additional brief piece of text distinguishing our streams from more extreme geothermal systems “Note, the warming here is indirect through the bedrock, which is distinct to that in, for example, Yellow Stone Park which experiences extreme water chemistries” on lines 76 to 78.

My specific comments are all minor:

Line 107 - grammar

We have revised this legend completely to address both this grammatical point and the editorial request to define our error bars, in this case “credible intervals”. This part now reads:

“Despite regional differences in temperature sensitivities (inset shows posterior densities for estimated region-level activation energies), CH_4 emissions (*n*=148 for 51 streams) increased with temperature across all regions on average (black line, symbols are streams shaped and coloured by region; grey shading shows 95% credible intervals). This increase was faster than expected from physical effects alone (in blue).” See revise legend for Figure 1 under Figure Legends.

Line 148 - odd phrasing - not an example - this is the beginning of your mechanistic explanation

True, probably cleanest to have no connecting phrase at all. See line 131.

Line 181 - hydrogenotroph, singular

Corrected, see line 149.

Line 181 - this is not an example, so reword

Point taken, and revised to “Specifically, as the proportion….” See line 149.

Line 251 - new subheading here?

Assuming it aligns with the NCLIM format we have added “Conclusions” before the last paragraph. See line 205.

Line 323-4 – grammar

Inserted “were” before “clearly”. See revised Extended Data Figure 3 legend in the Inventory of Supporting Information.

Line 688 - slope, not slop

Corrected to slope, see line 698.

Line 385 - scale bar is cut off at top of figure

Figure replaced accordingly. See new Extended Data Figure 6 on.

Reviewer #2 (Comments for the Author):

The authors have done a great and thorough job in the answers to my comments and in this revision. This work is novel, interesting and highly important. Thank you.

I have only two very minor comments:

Line 78. “temperature was a key environmental factor”. In what was temp the key environmental factor?

True, edited to “temperature is a key environmental factor explaining structure in the data”. See line 81.

Line 641. Should be probably MUSCLE aligner? Not MUCLE

Thanks, corrected to MUSCLE. See line 650.

Jackson, M. C. and others 2024. Regional impacts of warming on biodiversity and biomass in high latitude stream ecosystems across the Northern Hemisphere. Communications Biology **7:** 316.

O'gorman, E. J. and others 2014. Climate change and geothermal ecosystems: natural laboratories, sentinel systems, and future refugia. Glob Chang Biol **20:** 3291-3299.
